# Reversible synaptic adaptations in a subpopulation of murine hippocampal neurons following early-life seizures

**DOI:** 10.1172/JCI175167

**Published:** 2024-01-16

**Authors:** Bo Xing, Aaron J. Barbour, Joseph Vithayathil, Xiaofan Li, Sierra Dutko, Jessica Fawcett-Patel, Eunjoo Lancaster, Delia M. Talos, Frances E. Jensen

**Affiliations:** Department of Neurology, Perelman School of Medicine, University of Pennsylvania, Philadelphia, Pennsylvania, USA.

**Keywords:** Neuroscience, Epilepsy, Mouse models, Seizures

## Abstract

Early-life seizures (ELSs) can cause permanent cognitive deficits and network hyperexcitability, but it is unclear whether ELSs induce persistent changes in specific neuronal populations and whether these changes can be targeted to mitigate network dysfunction. We used the targeted recombination of activated populations (TRAP) approach to genetically label neurons activated by kainate-induced ELSs in immature mice. The ELS-TRAPed neurons were mainly found in hippocampal CA1, remained uniquely susceptible to reactivation by later-life seizures, and displayed sustained enhancement in α-amino-3-hydroxy-5-methyl-4-isoxazolepropionic acid (AMPA) receptor–mediated (AMPAR-mediated) excitatory synaptic transmission and inward rectification. ELS-TRAPed neurons, but not non-TRAPed surrounding neurons, exhibited enduring decreases in *Gria2* mRNA, responsible for encoding the GluA2 subunit of the AMPARs. This was paralleled by decreased synaptic GluA2 protein expression and heightened phosphorylated GluA2 at Ser880 in dendrites, indicative of GluA2 internalization. Consistent with increased GluA2-lacking AMPARs, ELS-TRAPed neurons showed premature silent synapse depletion, impaired long-term potentiation, and impaired long-term depression. In vivo postseizure treatment with IEM-1460, an inhibitor of GluA2-lacking AMPARs, markedly mitigated ELS-induced changes in TRAPed neurons. These findings show that enduring modifications of AMPARs occur in a subpopulation of ELS-activated neurons, contributing to synaptic dysplasticity and network hyperexcitability, but are reversible with early IEM-1460 intervention.

## Abstract 2

Early seizures induce synaptic adaptations in specific neuronal subpopulations.

## Introduction

Both clinical and experimental evidence suggest that seizures in early life can be associated with long-lasting cognitive and behavioral deficits (1–6), yet the mechanisms of these long-term changes remain unclear and no specific therapies are in clinical use (7). Neurodevelopmental disorders associated with autism and intellectual disability often involve early-life seizures (ELSs) (8–10). By definition, ELSs occur in developing neuronal networks during a critical period of synaptogenesis (7), and we have shown they cause rapid activity-dependent modifications of excitatory glutamate receptors, in particular the α-amino-3-hydroxy-5-methyl-4-isoxazolepropionic acid (AMPA) glutamate receptor (AMPAR) subtype (6, 11–13). These early changes in AMPARs can impair synaptic and critical period plasticity, resulting in long-term social and learning deficits and seizure susceptibility in rodent models (6, 7, 14–17). Importantly, a specific form of the AMPARs, those lacking the GluA2 subunit, has been linked to modifications in plasticity in health and disease across the lifespan and has been shown to be specifically upregulated under conditions of ELS (11–13, 18–20).

GluA2-lacking AMPARs have higher channel conductance and enhanced calcium (Ca^2+^) influx, which are associated with greater postsynaptic depolarization compared with GluA2-containing receptors (19, 21, 22). Together, these have been implicated in contributing to heightened excitability and epileptogenesis in the immature brain (13, 23, 24). ELS-induced changes in AMPAR function may also contribute to ELS-associated developmental dysregulation and long-term cognitive impairment. Supporting their critical role, we and others have shown that in vivo treatment with IEM-1460, an adamantine derivative and specific open channel blocker of GluA2-lacking AMPARs (25), can rescue seizure-associated impairment of network plasticity, seizure susceptibility, and behavioral deficits (23, 26, 27).

Despite the persistent cognitive deficits seen experimentally and clinically, previous studies evaluating long-term effects of ELSs have not been able to track changes in gene and protein expression or function at the single neuron level in later stages of life. Recent evidence from human tissue biopsies and rodent seizure models suggests that seizures can elicit heterogeneous responses within distinct neuronal subpopulations and some alterations in synaptic plasticity can occur over days and weeks after a seizure (24, 28–33). However, it is not known whether these neuronal subpopulations drive the long-lasting behavioral changes and impaired network plasticity associated with ELSs and prior ELS work has been hindered by the lack of ability to track the evolution of changes to single neurons throughout the lifespan. Given these data, we hypothesized that ELSs would induce heterogeneous neuronal activation, and that this initial seizure event may cause long-lasting pathological transcriptional, protein, and synaptic changes specifically in neurons involved in ELSs.

To test our hypothesis, we used a well-established FosTRAP (targeted recombination in active population) mouse line, which allowed us to permanently label cells that were activated by ELSs (TRAPed) (34, 35). Subsequently, we investigated and compared ELS-TRAPed and surrounding non–ELS-activated CA1 hippocampal neurons for cell-specific synaptic responses, transcriptional and posttranslational modifications of select synaptic proteins, and differential activation during subsequent later-life seizure (LLS) events. First, we found that ELS induction on postnatal day 10 (P10) activates only a subset of hippocampal neurons, and that these neurons were more susceptible to reactivation by later-life-induced seizures compared with surround non-TRAPed neurons. These ELS-TRAPed neurons exhibited long-lasting decreases in *Gria2* mRNA levels and posttranslational modification of the GluA2 subunit protein, as well as altered AMPAR currents and impaired long-term potentiation (LTP) and long-term depression (LTD) at P30. Furthermore, the current study shows that early postseizure IEM-1460 treatment prevented the long-term changes in AMPAR function and synaptic plasticity in TRAPed neurons. Understanding the cell-specific mechanisms by which seizures lead to cognitive and behavioral deficits is crucial for developing therapeutic strategies in this clinical space where no current cure exists.

## Results

### ELSs differentially activate specific neuronal populations.

**Figure 1:**
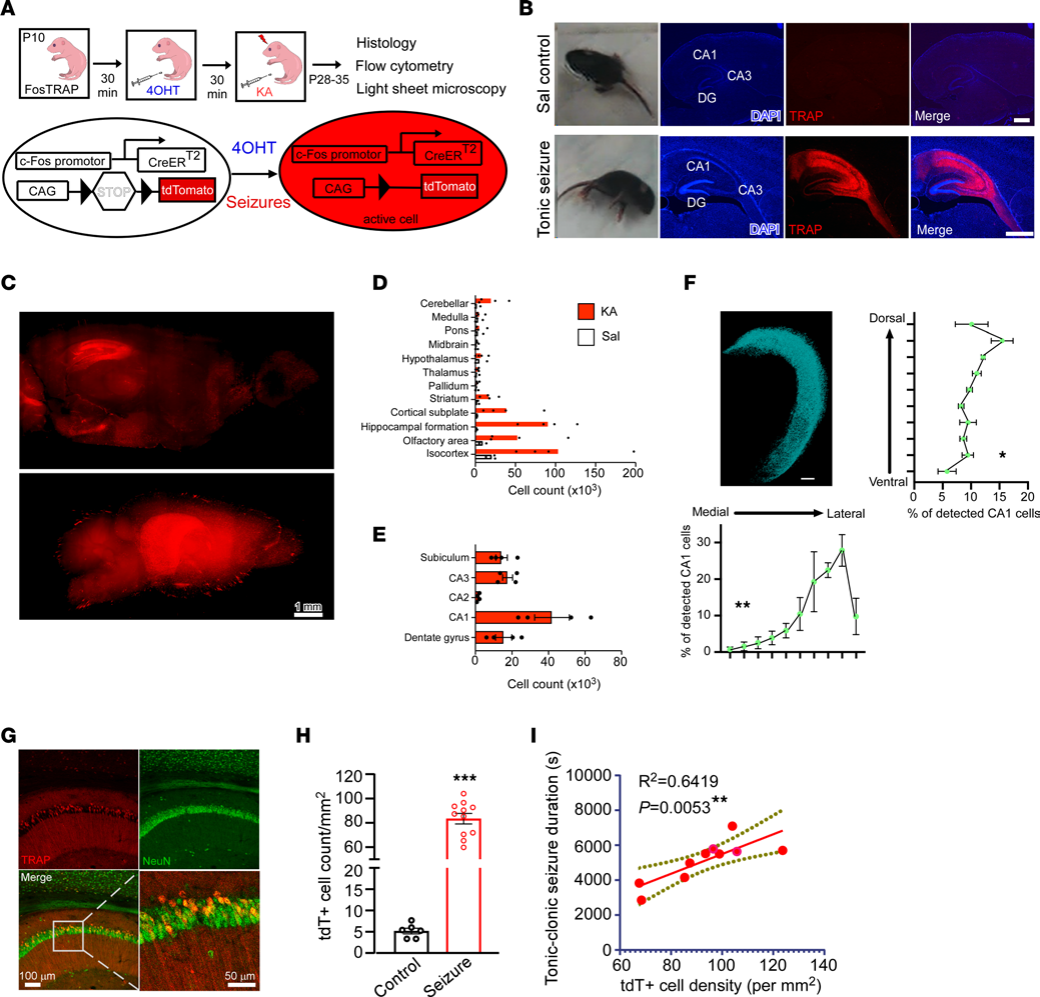
FosTRAP permanently and selectively labels ELS-activated cells. (**A**) Schematic of experimental paradigm and TRAPing of neurons active during ELSs by paring 4-OHT and KA (2 mg/kg, i.p.). (**B**) Representative images of sagittal sections from FosTRAP mice injected with saline (Sal controls, upper) and KA (tonic-clonic seizures, lower). TRAPed cells expressing tdTomao (tdT, red) are enriched in the hippocampus. Scale bars: 500 μm. (**C**–**F**) Light-sheet fluorescence microscopy (LSFM) of ELS-TRAPed mouse brain (see Supplemental Video 1). (**C**) LSFM images of sagittal maximum intensity projection across 10 planes (top) and 3D reconstruction of KA-treated TRAPed hemisphere (bottom). Scale bar: 1 mm. (**D**) Results from the brain mapping pipeline displaying cell counts from Sal- and KA-treated mice broken down into broad regions according to the Allen Brain Atlas hierarchy. (**E**) Cell counts from hippocampal subregions. (**F**) 3D reconstruction of all detected CA1 cells from a KA-treated TRAP hemisphere (top left). Cellular distribution along the dorsal-ventral (right) and medial-lateral axes (bottom) from KA-treated TRAP mice. CA1 tdT^+^ locations were quantified along the dorsal/ventral and lateral/medial axes. *P < 0.05, **P < 0.01 by linear regression (*n* = 4 Sal and 4 KA). (**G**) Representative confocal images of hippocampal CA1 region immunolabeled for NeuN showing ELS-TRAPed neurons. Images in **G** are shown again in Supplemental Figure 4A. Scale bars: 100 μm (top row and bottom left) and 50 μm (bottom right). (**H**) Quantitative confocal imaging analysis showed a higher density of TRAPed (tdT^+^) cells in the CA1 region from KA-treated mice compared with Sal controls. ***P < 0.001 by Mann-Whitney *U* test (*n* = 6 Sal and 11 KA). (**I**) Linear regression of tonic-clonic seizures duration and density of TRAPed cells (*n* = 10 mice) in CA1 region. The dashed lines define the 95% confidence interval. *R*^2^ = 0.6419, **P < 0.01. Data expressed as mean ± SEM.

Multiple converging lines of evidence increasingly suggest that seizures at their onset induce heterogeneous responses within distinct neuronal subpopulations (23, 29). To permanently label the active cells during seizures, we used FosTRAP-transgenic mice (hereafter, FosTRAP mice) (34, 35) in combination with a well-established chemoconvulsant kainic acid (KA) mouse model of ELS (36, 37). These mice express Cre recombinase (CreER) under the control of an activity-dependent endogenous c-Fos promoter, which induces the recombination of the double-floxed inverse open reading frame in the presence of 4-hydroxytamoxifen (4-OHT, 50 mg/kg, i.p.), resulting in permanent tdTomato (tdT) expression in the cell population activated during seizures (Figure 1A).

Following 4-OHT administration on P10, tonic-clonic seizures were induced by administration of KA (2 mg/kg i.p.) to label activated (TRAPed) populations so that transcript, protein, and functional changes in ELS-TRAPed neurons could be sampled at various intervals in later life from P14 to P30–P35 days (Figure 1A). To confirm the absence of cell death following KA-induced ELSs at P10, a subset of mice was sacrificed 24 hours after seizure for Fluoro-Jade B (FJB) staining, which indeed demonstrated no overt neuronal death (Supplemental Figure 1; supplemental material available online with this article; https://doi.org/10.1172/JCI175167DS1), consistent with previous studies (16–18, 38, 39). Hippocampal tissue examination 18–25 days after seizure (P28–P35) showed substantially more tdT^+^ cells in the KA-treated mice than in the saline-treated (Sal-treated) littermates, exhibiting only minimal background labeling (Figure 1B). Next, we used brain clearing and light-sheet fluorescence microscopy (LSFM) to further map the brain-wide distribution of ELS-activated cellular ensembles 21 days after ELS to obtain single-cell resolution images of intact hemispheres of FosTRAP mice (Figure 1, C–F, and Supplemental Table 1). As expected, ELS cerebral hemispheres showed dramatic increases in tdT^+^ cells compared with control animals across all major brain regions, with the isocortex and hippocampus displaying the highest levels of activation (Figure 1D and Supplemental Figure 2), including robust activation of hippocampal CA1 (Figure 1E), an area in which we have previously demonstrated enduring network changes after ELS (12, 13, 40). Linear regression analysis demonstrated significant increases in the density of tdT^+^ cells in dorsal and lateral portions of the CA1 (*n* = 4 dorsal/ventral, *P* < 0.05; *n* = 4 medial/lateral, *P* < 0.01; Figure 1F), suggesting increased susceptibility of this region to ELSs.

Confocal imaging analysis of the CA1 region confirmed increased tdT labeling following ELSs (Figure 1G) compared with basal levels of tdT^+^ cells in control mice (Figure 1H). Moreover, the density of tdT^+^ cells in CA1 positively correlated with seizure duration (*R*^2^ = 0.6419, *P* < 0.01; Figure 1I), indicating that the ELS-TRAPed cell number is tightly linked to an individual animal’s seizure severity. Importantly, mice that did not experience tonic-clonic seizures following KA administration exhibited limited tdT labeling in CA1 compared with those with tonic-clonic seizures (Supplemental Figure 3), suggesting that the activation of CA1 neurons was a consequence of seizures rather than the direct result of KA injection.

To further characterize which cell types were TRAPed in area CA1 of the hippocampus during ELSs, we quantified the colocalization of ELS-TRAPed cells and several known neuronal and glial markers (Supplemental Figure 4). We found that 95.3% ± 1.0% of TRAPed cells (tdT^+^) were neurons, as indicated by the colocalization with NeuN (Figure 1G and Supplemental Figure 4A), and their pyramidal neuron morphology, such as those located in the stratum pyramidale. The remainder of tdT^+^ cells were identified as GABAergic interneurons (Supplemental Figure 4B), astrocytes (Supplemental Figure 4C), and microglia (Supplemental Figure 4D) by immunostaining for GAD67 (2.0% ± 1.1%), GFAP (6.9% ± 1.1%), and Iba-1 (1.0% ± 0.6%) (Supplemental Figure 4E). Together, these results suggest that ELS-activated neurons in the CA1 region are predominantly pyramidal neurons.

### ELS-TRAPed neurons are preferentially reactivated by LLSs.

**Figure 2:**
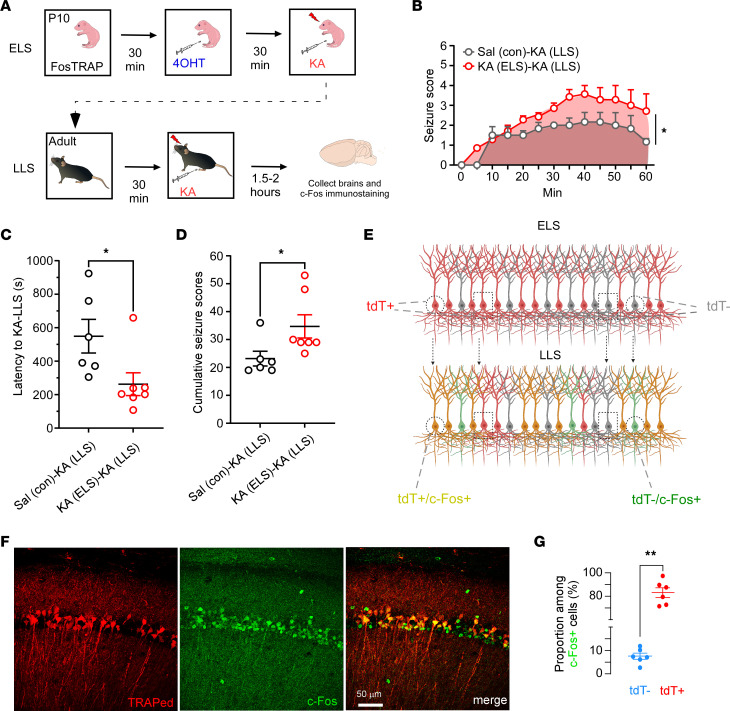
ELS-TRAPed cells are preferentially reactivated by later life seizures (LLSs). (**A**) Schematic illustration of the experimental procedure. At P10, mice were TRAPed by pairing 4-OHT with KA. At adulthood, both groups received a second seizure (LLS) using KA (15 mg/kg), and mice were immediately euthanized for c-Fos immunostaining. The experimental groups are referred to as KA (ELS) and Sal (con) for the P10 experiment, and KA (ELS) + KA (LLS) (red in **B**–**E**) and Sal (con) + KA (LLS) (gray in **B**–**E**) for the second seizure experiment at adulthood. (**B**) Time course of seizure score following LLS-KA treatment (left). The mice with prior ELS (*n* = 7 mice) showed significantly higher seizure severity, assessed by area under the curve (AUC), than those without ELS (*n* = 6 mice): 146.8 (95% CI 117.7 to 175.9) versus 95.42 (95% CI 71.76 to 119.1). *P < 0.05 by unpaired, 2-tailed *t* test. (**C**) Latency to LLSs showing mice with prior ELSs reaching the first behavioral seizure significantly faster than those without ELSs. *P < 0.05 by Mann-Whitney *U* test. (**D**) Cumulative seizure scores indicate that mice with ELS exhibit more severe seizures during LLS. *P < 0.05 by Mann-Whitney *U* test. (**E**) Schematic representation of distinct cell populations responding to ELS and LLS. (**F**) Example confocal images showing CA1 neurons labeled with tdT (ELS at P10) and c-Fos (LLS at adulthood) labeled with Alexa Fluor 488 reveal that c-Fos activation induced by LLS occurred primarily in previously activated tdT^+^ ELS-TRAPed neurons. Scale bar: 50 μm. (**G**) Quantification of tdT^–^ and tdT^+^ proportions among c-Fos^+^ cells (*n* = 6 mice). **P < 0.01 by Mann-Whitney *U* test. Data expressed as mean ± SEM.

The brains of animals that experienced ELSs are more prone to seizures and exhibit long-term hyperexcitability, even into adulthood (17, 41, 42). While hippocampal CA1 is one of the major structures involved in ELS, it remains unclear to what extent neurons activated by the original ELS contribute to network hyperexcitability and LLSs. To explore this question, FosTRAP mice underwent the ELS protocol at P10, and 2 to 3 months later (P70–P100), a second seizure was induced with a single KA injection (15 mg/kg, i.p.) (Figure 2A). Consistent with our prior reports (11, 39, 43), we found that the mice with prior ELS [KA (ELS) + KA (LLS)] showed increased seizure severity compared with the P10 Sal controls [Sal (con) + KA (LLS)] over a 1-hour period (Figure 2B). The adult mice with prior ELS also exhibited shorter latency to the first behavioral seizure (Figure 2C) and higher cumulative seizure scores (Figure 2D). The mice were then sacrificed 2 hours after LLS to obtain samples, and sections were immunostained for cFos with an Alexa Fluor 488–conjugated secondary antibody so that double labeling with ELS-induced tdT^+^ could be assessed (Figure 2E). Immunostaining revealed that 89.3% ± 6.8% (*n* = 6) of Alexa Fluor 488–tagged c-Fos–positive cells within CA1 due to activation during LLS were positive for tdT (Figure 2, F and G). The observation that ELS-TRAPed neurons were predominantly reactivated by LLS indicates they remain preferentially susceptible to seizures compared with surrounding neurons and thus may represent a seizure engram contributing to epileptogenesis.

### ELSs induce persistent enhanced AMPAR-mediated synaptic transmission only in TRAPed neurons.

**Figure 3:**
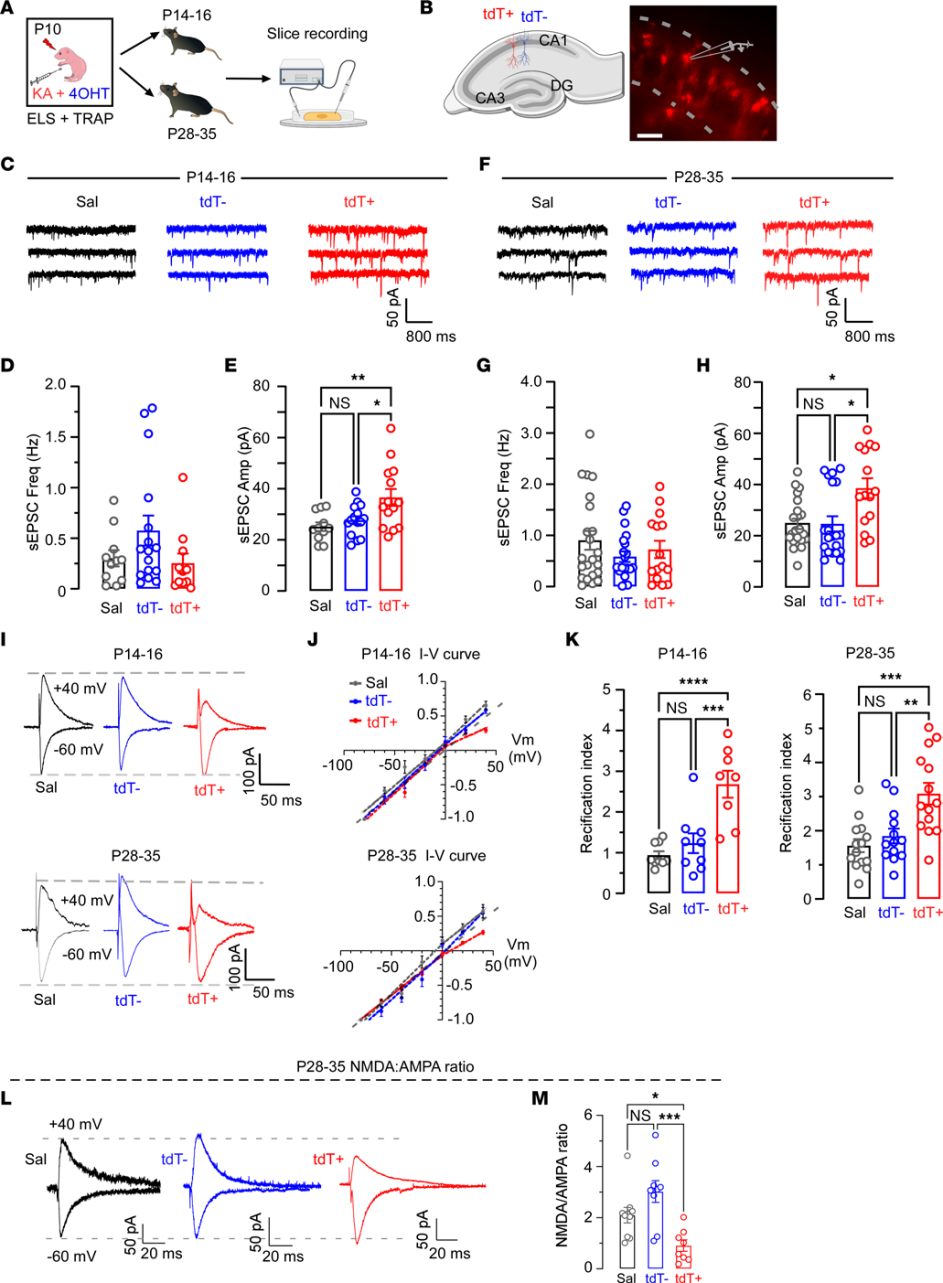
Persistent increased excitatory synaptic transmission onto ELS-TRAPed cells after ELS. (**A**) Schematic of slice recordings. (**B**) Left: Schematic of the tdT^–^ and tdT^+^ neurons in CA1. Right: DIC image of TRAPed neurons in CA1. Scale bar: 50 μm. (**C**) Examples of sEPSCs recorded from Sal (*n* = 11 neurons/6 mice), tdT^–^ (*n* = 16 neurons/8 mice), and tdT^+^ (*n* = 14 neurons/8 mice) neurons at P14–P16. (**D**) Mean frequency and (**E**) mean amplitude of sEPSCs from Sal, tdT^–^, and tdT^+^ neurons at P14–P16. *P < 0.05, **P < 0.01 by 1-way ANOVA followed by Tukey’s test. (**F**) Examples of sEPSCs recorded from Sal, tdT^–^, and tdT^+^ neurons at P28–P35. (**G**) Mean frequency and (**H**) mean amplitude of sEPSCs from Sal (*n* = 22 neurons/8 mice), tdT^–^ (*n* = 20 neurons/8 mice), and tdT^+^ (*n* = 15 neurons/8 mice) at P28–P35. *P < 0.05 by Kruskal-Wallis test followed by Dunn’s test. (**I**) Example EPSCs elicited at –60 mV and +40 mV from Sal, tdT^–^, and tdT^+^ neurons at P14–P16 (top) and P28–P35 (bottom). (**J**) I-V curves of EPSCs from Sal (*n* = 9 neurons/4 mice), tdT^–^ (*n* = 9 neurons/4 mice), and tdT^+^ (*n* = 8 neurons/4 mice) at P14–P16 (top) and P28–P35 (bottom) (Sal, *n* = 14 neurons/5 mice; tdT^–^, *n* = 13 neurons/5 mice; and tdT^+^, *n* = 14 neurons/5 mice). (**K**) Increased rectification of EPSCs was detected in tdT^+^ neurons at P14–P16 (left) and at P28–P35 (right). **P < 0.01, ***P < 0.001, ****P < 0.0001 by 1-way ANOVA followed by Tukey’s test. (**L**) Sample traces of AMPAR EPSCs at –60 mV and NMDAR EPSCs at +40 mV were recorded at P28–P35. (**M**) Quantification of the NMDA/AMPA ratio (Sal, *n* = 9 neurons/4 mice; tdT^–^, *n* = 8 neurons/4 mice; tdT^+^, *n* = 8 neurons/4 mice). *P < 0.05, ***P < 0.001 by 1-way ANOVA followed by Tukey’s test. Data expressed as mean ± SEM.

Given the specificity of the neuronal activation during ELS and susceptibility to LLS-induced activation, we compared synaptic transmission onto tdT^+^ neurons to that of surrounding tdT^–^ neurons as well as onto neurons in Sal-treated controls. After ELS at P10, horizontal brain slices containing the hippocampus were prepared and whole-cell patch clamp recordings were performed at an early (P14–P16) or later (P28–P35) time point (Figure 3A), allowing selective recordings from tdT^+^ and tdT^–^ CA1 pyramidal neurons (Figure 3B).

We first examined AMPAR-mediated spontaneous excitatory postsynaptic currents (sEPSCs), which were recorded in the presence of picrotoxin to block GABA_A_-mediated inhibitory synaptic currents (Figure 3, C and F). While the sEPSC frequencies of tdT^+^, tdT^–^, and neurons from control mice treated with Sal at P10 were comparable in either age group (Figure 3, D and G), it is notable that the sEPSC amplitudes were significantly larger in tdT^+^ neurons compared with tdT^–^ in the P10 KA-treated group, and compared with neurons from Sal-treated mice at both P14–P16 (Figure 3E) and P28–P35 (Figure 3H), consistent with a persistent postsynaptic mechanism for the enhanced synaptic transmission. Importantly, passive (resting membrane potential and membrane resistance) and active (action potential amplitude, duration, and threshold) intrinsic properties of these neurons were measured and there were no significant differences among tdT^+^ and tdT^–^ neurons in the KA-treated mice and those from the Sal-treated controls (data not shown).

We and others have found that the presence of GluA2-lacking, Ca^2+^-permeable AMPARs following seizures contribute to persistent synaptic dysregulation (6, 12, 13, 23, 44–47), but it was unclear whether this was specific to ELS-activated neurons. GluA2-lacking AMPARs can be detected by inwardly rectifying currents, showing reduced conductance at positive membrane potentials (19). We examined evoked AMPAR-mediated transmission at P14–P16 or P28–P35 to determine whether there was a selective change in rectification in tdT^+^ neurons (Figure 3I). The cells were held at potentials from –80 to +40 mV in –20 mV increments and the evoked currents at each holding potential were recorded. The tdT^–^ neurons and those from Sal control mice showed a linear current-voltage (I-V) curve (Figure 3J), consistent with GluA2-containing AMPARs, normally observed at mature synapses in the hippocampus (19, 48). In contrast, we observed significantly increased rectification of AMPAR EPSCs selectively in the tdT^+^ neurons compared with tdT^–^neurons in slices from ELS mice, manifested by a nonlinear I-V relationship and higher rectification index (EPSC_−60mV_/EPSC_+40mV_) (Figure 3, J and K), characteristic of an increased population of GluA2-lacking AMPARs. These results suggest that ELS induces a selective persistence of GluA2-lacking AMPARs in synapses on tdT^+^ CA1 neurons across the postnatal developmental period into early adulthood.

Activation of GluA2-lacking AMPARs at synapses on a single neuron has been reported to attenuate the *N*-methyl-D-aspartate receptor–mediated (NMDAR-mediated) current via modulating intracellular Ca^2+^ (49). Given the persistent changes in AMPAR function, we tested whether NMDARs are functionally impaired in the ELS-TRAPed cells by measuring the ratio of the amplitude of NMDAR- to AMPAR-mediated EPSCs (NMDA/AMPA ratio) recorded in tdT^+^ and tdT^–^ neurons from FosTRAP mice with ELS and cells from P10 Sal-treated mice as control. Consistently, slices removed at P28–P35 after ELS at P10 showed a significantly lower NMDA/AMPA ratio in tdT^+^ neurons than that of surrounding tdT^–^ neurons and those from Sal (no seizure) controls (Figure 3, L and M).

### Evidence of persistent and selective GluA2 downregulation in TRAPed CA1 neurons after ELS at P10.

To determine the mechanism of the apparent downregulation of GluA2 as indicated by the electrophysiological studies, we examined hippocampi for evidence of changes in gene and/or protein expression in response to ELS. Since our prior research identified lower levels of GluA2 expression of AMPARs in neurons in this region following ELS (13, 18), we sought to determine whether the relative expression of *Gria1* and *Gria2* transcripts, encoding for GluA1 and GluA1 respectively, were altered specifically in ELS-TRAPed neurons.

**Figure 4:**
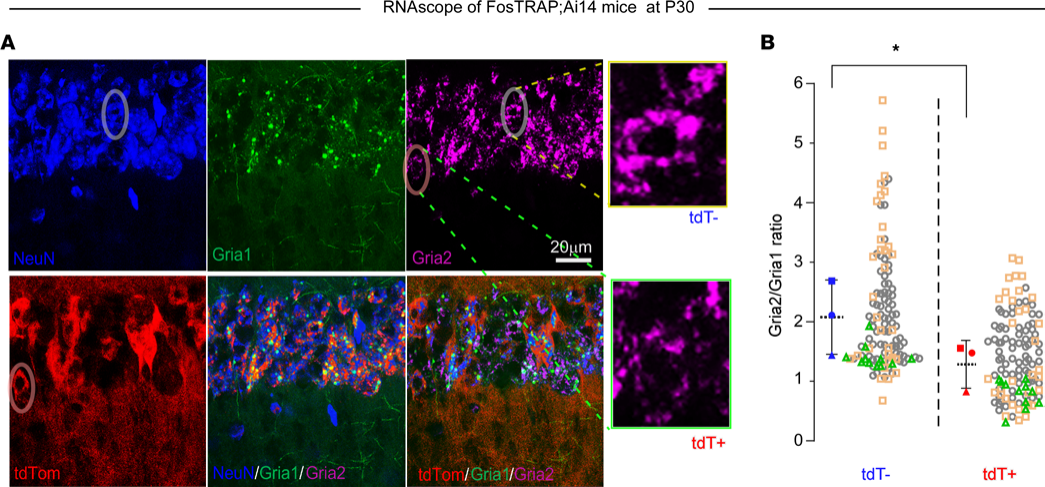
RNAscope at P30 identifies cell type–specific transcriptional dysregulation of AMPAR subunits in TRAPed CA1 neurons after ELS. (**A**) Confocal images of an example hippocampal slice showing colocalization of NeuN, TRAP (tdTomato^+^ [tdT^+^]), and mRNA expression of *Gria1* and *Gria2* using RNAscope. Scale bar: 20 μm. (**B**) Summary graph showing that tdT^+^ neurons (*n* = 136 cells) expressed a lower *Gria2*/*Gria1* ratio compared with the surrounding tdT^–^ neurons (*n* = 148 cells). Cell-level data are color coded (1 color represents one animal) according to each biological sample and the sample-level means are compared between groups (*n* = 3 mice/group). *P* < 0.05 by 2-tailed, paired *t* test. Data expressed as mean ± SEM.

We first examined the mRNA expression of these transcripts by RNAscope in situ hybridization (ISH) at a single-cell level in FosTRAP mice. Our results showed that in hippocampi removed on P30 following ELS at P10, both *Gria1* and *Gria2* were highly expressed in the pyramidal cell layer of CA1, with only a few *Gria1* gene transcripts detected in the stratum radiatum (Figure 4A). Consistent with the electrophysiological evidence for a selective loss of GluA2, we found that the ratio of *Gria2/Gria1* expression in CA1 pyramidal neurons was significantly lower in the tdT^+^ neurons compared with surrounding tdT^–^ neurons (Figure 4B). These findings suggest that the ELS-TRAPed neurons are enriched in GluA2-lacking AMPARs, which are known to lead to hyperactivity, in part due to excessive influx of Ca^2+^ (22, 50). Importantly, these results suggest that ELSs can lead to enduring changes in AMPAR subunit gene expression specific to TRAPed neurons.

**Figure 5:**
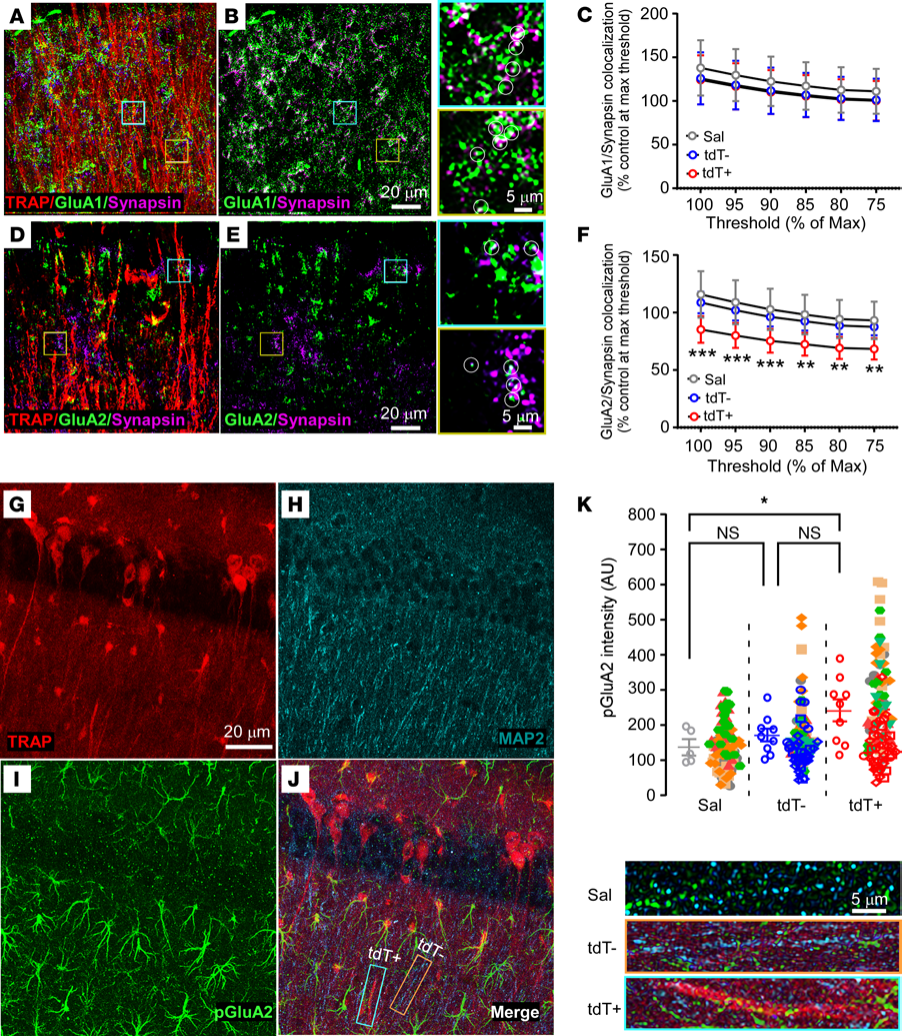
Altered protein expression and phosphorylation status of AMPAR subunits in the ELS-TRAPed neurons. (**A** and **B**) Representative confocal images of CA1 radiatum from an ELS-TRAPed mouse. (**A**) Merged image showing the colocalization of TRAPed (red) dendrites, synapsin (synapse marker, purple), and GluA1 (green). (**B**) Synapsin and GluA1 merged image (left) shows the colocalization of synapsin and GluA1 from tdT^+^ and tdT^–^ dendrites. Right panels represent higher-magnification images of tdT^+^ (blue box) and tdT^–^ (yellow) dendrites. (**C**) Quantitative analysis showed no significant change in GluA1/synapsin colocalization in tdT^+^ dendrites compared to Sal and tdT^–^ by 2-way ANOVA (Sal, *n* = 7; ELS-TRAP, *n* =6). (**D** and **E**) Examples of confocal images of CA1 radiatum from an ELS-TRAPed mouse. (**D**) Merged image showing the colocalization of TRAP (red) dendrites, synapsin (purple), and GluA2 (Green). (**E**) Synapsin and GluA2 merged images show the colocalization of synapsin and GluA2 from tdT^–^ and tdT^+^ dendrites. Right panels represent higher-magnification images of tdT^+^ (blue box) and tdT^–^ (yellow box) dendrites. Scale bars (**A**–**E**): 20 μm and 5 μm (zoomed-in images on right). (**F**) Quantification of GluA2/synapsin showing lower colocalization in tdT^+^ dendrites compared with Sal and tdT^–^ dendrites. **P < 0.01, ***P < 0.001 by 2-way ANOVA followed by Tukey’s test. Sal, *n* = 7 mice; ELS-TRAP, *n* = 6 mice. (**G**–**J**) Representative confocal images of CA1 depicting TRAPed cells expressing tdTomato (**G**) with MAP2 (dendrite markers, **H**) and pGluA2-S880 (**I**) staining. The merged image (**J**) shows the colocalization of tdT^–^ (blue box) and tdT^+^ (orange box) dendrites. High-magnification images (bottom right) and summary graph (**K**) demonstrate higher pGluA2-S880 levels in tdT^+^ dendrites. Scale bars: 20 μm (**G**–**J**) and 5 μm (**K**). Sal, *n* = 5; ELS-TRAP, *n* = 9. Cell-level data are color coded (1 color represents 1 animal), and the sample-level means from pooled cell-level data are compared between groups. *P < 0.05 by 1-way ANOVA followed by Tukey’s test. Data expressed as mean ± SEM.

In addition to altering gene expression (51), seizures can lead to long-lasting changes in synaptic plasticity, hyperexcitability, and seizure susceptibility in the brain by inducing activity-dependent posttranslational modifications of synaptic GluA1 and GluA2 subunit proteins (11, 12). We thus examined whether there were any persistent cell-specific changes in the expression of these synaptic proteins within ELS-TRAPed cells at P30. We performed double staining of GluA1 (Figure 5, A–C) or GluA2 (Figure 5, D–F) with the presynaptic marker synapsin in CA1 stratum radiatum and stratum lacunosum-moleculare, where apical dendrites of pyramidal cells are located (52). Indeed, GluA2-synapsin colocalization was significantly decreased in tdT^+^ dendrites from P30 mice after P10 KA-induced seizures compared with the tdT^–^ surrounding neurons and those from Sal-treated (no ELS at P10) controls (Figure 5, D–F), indicating a selective loss of synaptic GluA2 expression in the ELS-TRAPed neurons. Notably, there was no significant difference in the immunostaining intensity of synaptic GluA1 expression (colocalized GluA1/synapsin puncta) among Sal control, or P10 KA-treated tdT^+^ and tdT^–^ neuronal dendritic processes (Figure 5, A–C).

GluA2 levels are subject to tight regulation through posttranslational phosphorylation, and elevated phosphorylation of GluA2 at Ser880 is associated with the promotion of AMPAR endocytosis and the formation of GluA2-deficient AMPARs (20). We measured the S880-phosphorylated GluA2 (p-GluA2-S880) in apical dendrites (stained for MAP2) of CA1 neurons at P30 and found that these levels were higher in tdT^+^ dendrites than in the Sal group (Figure 5, G–K). These results suggest that ELSs induce a persistent downregulation of synaptic GluA2, but not GluA1, via an activity-dependent subunit removal selectively in ELS-activated neurons.

### ELS facilitates premature synapse unsilencing in TRAPed neurons.

**Figure 6:**
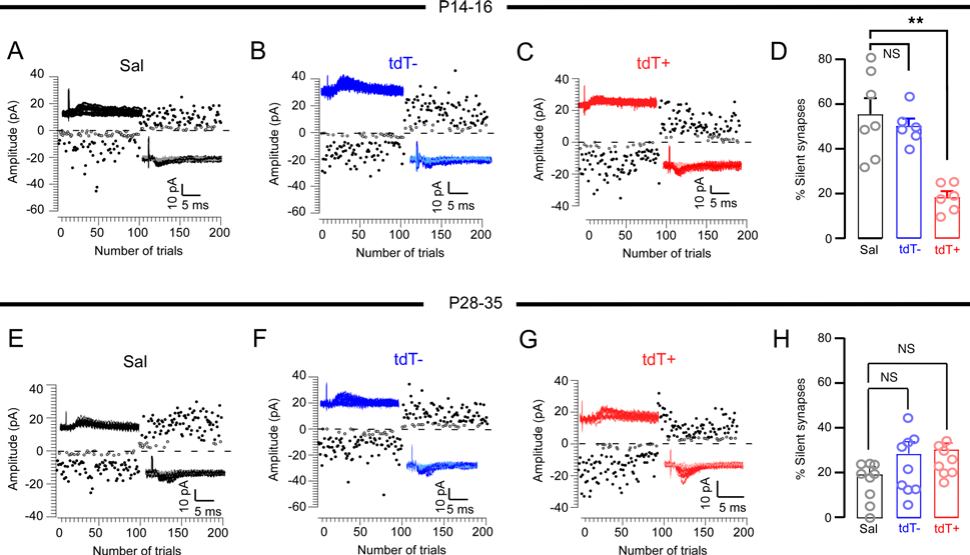
Accelerated developmental loss of silent synapses at ELS-TRAPed CA1 neurons. Representative traces and plots of minimally evoked EPSCs in Sal (black), tdT^–^ (blue), and tdT^+^ (red) CA1 neurons at P14–P16 (**A**–**D**) and P28–P35 (**E**–**H**). Quantification of the percentage of silent synapses at Sal, tdT^–^, and tdT^+^ neurons. EPSCs elicited at –60 mV and +40 mV (**D** and **H**). There was a significantly lower percentage of silent synapses at P14–P16 (Sal, *n* = 7 from 3 mice; tdT^–^, *n* = 6 from 3 mice; and tdT^+^, *n* = 6 from 3 mice. **P < 0.01 by Kruskal-Wallis test followed by Dunn’s test), but not at P28–P35 (Sal, *n* = 9 from 3 mice; tdT^–^, *n* = 9 from 8 mice; and tdT^+^, *n* = 8 from 3 mice). Data expressed as mean ± SEM.

Disruptions in AMPAR gene expression and trafficking can perturb the typical early postnatal development of AMPARs, potentially leading to impaired synaptic plasticity. During early stages of postnatal development, NMDAR-only synapses lacking functional AMPARs are silent but become unsilenced when they acquire AMPARs through coordinated activity and experience, contributing to the refinement and adjustment of neural circuits (6, 53, 54). Relevant to the clinical observation of increased risk of neurodevelopmental cognitive deficits after ELS, we have previously shown that ELS reduces the fraction of silent synapses and which results in the occlusion of LTP of CA1 neurons (55). Consequently, ELS prematurely alters silent synapses due to activity-dependent trafficking of receptors, impairing and occluding subsequent critical period plasticity (6, 55). To better understand how ELSs might disrupt synaptic plasticity during development, we investigated the role of silent synapse loss in the synaptic hyperexcitability observed in ELS-TRAPed neurons. We examined silent synapses in ELS-TRAPed tdT^+^ neurons and surrounding tdT^–^ neurons compared with cells from P10 Sal-treated animals by using our published protocols of minimally evoked EPSCs and failure rates, measuring the ratio of silent to functional synapses (6, 55). Notably, we observed that the proportion of silent synapses in the tdT^+^ neurons significantly decreased 4 to 6 days after ELS, while there were no changes in the tdT^–^ surrounding neurons (Figure 6, A–D). To investigate whether the synaptic unsilencing continues into later life, we evaluated the fraction of silent synapses at P28–P35. Given these recordings were performed in mature networks (P30), the expression of silent synapses was low and comparable among tdT^+^, tdT^–^, and Sal groups (Figure 6, E–H), consistent with prior reports that few silent synapses exist after synapse maturation (6, 55). Taken together, these results indicate that ELS-induced neuronal activation accelerates the unsilencing of synapses and enhances AMPAR function selectively in ELS-TRAPed neurons.

### LTP and LTD are selectively disrupted in ELS-TRAPed neurons.

We have previously shown that ELS-induced unsilencing and strengthening of glutamatergic synapses in immature CA1 pyramidal networks can lead to impairment of subsequent LTP and LTD (6, 13, 55). To test whether the persistent ELS-evoked dysplasticity is specific to activated CA1 neurons, we compared LTP/LTD of tdT^+^ and tdT^–^ neurons in P28–P35 slices with prior ELS at P10 versus neurons in slices from Sal-treated (no seizure) mice.

**Figure 7:**
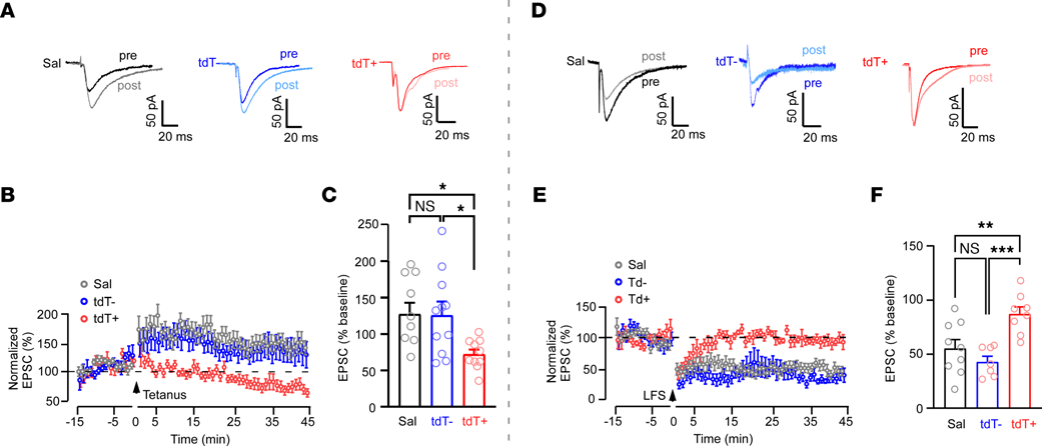
Diminished LTP and LTD in ELS-TRAPed CA1 neurons at P28–P35. (**A** and **B**) LTP paradigm, showing example traces and plots of evoked AMPA currents before and after paired LTP stimulation protocol (0.3 ms, 100 Hz, separated by 20 seconds) from Sal-treated mice, and tdT^–^ and tdT^+^ neurons from ELS-treated mice. Sal and tdT^–^ neurons exhibited a long-lasting increase in EPSC following tetanus (arrow), while tdT^+^ neurons did not show an increase in EPSC amplitude after stimulation. (**C**) Summary of LTP experiments showing significantly less LTP in tdT^+^ neurons compared with neurons from Sal-treated mice, or tdT^–^ neurons from ELS-treated mice (Sal, *n* = 10 neurons/4 mice; tdT^–^, *n* = 11 neurons/6 mice; tdT^+^, *n* = 11 neurons/6 mice). *P < 0.05 by Kruskal-Wallis test followed by Dunn’s test. (**D** and **E**) LTD paradigm, showing example traces and plots of evoked AMPA currents before and after long-frequency (5 Hz, 900 pulses) LTD stimulation (LFS) from Sal, tdT^–^, and tdT^+^ neurons. Sal and tdT^–^ neurons showed a long-lasting reduction in EPSC, but tdT^+^ neurons exhibited comparable EPSC amplitudes before and after long-frequency stimulation. (**F**) Summary of LTD experiments showing significantly less LTD in tdT^+^ neurons compared with tdT^–^ neurons from ELS mice or neurons from Sal-treated (no seizure) mice (Sal, *n* = 9 neurons/4 mice; tdT^–^, *n* = 8 neurons/5 mice; tdT^+^, *n* = 7 neurons/5 mice). **P < 0.01, ***P < 0.001 by 1-way ANOVA followed by Tukey’s test. Data expressed as mean ± SEM.

To induce LTP, we used a pairing protocol that involved 2 tetani (0.3 ms, 100 Hz, separated by 20 seconds) at the Schaffer collateral pathway, with CA1 neurons held at 10 mV (55). This LTP protocol led to a long-lasting increase in EPSC amplitude in tdT^–^ cells from the ELS group and neurons from the Sal (no-seizure) group, as expected. In contrast, tdT^+^ neurons did not show a significant increase in EPSC amplitude after the paired tetani stimulation, indicating a specific impairment of LTP in ELS-TRAPed neurons long after ELS (Figure 7, A–C). To induce LTD, we applied a low-frequency stimulation protocol (5 Hz, 900 pulses) with CA1 neurons held at –40 mV at the Schaffer collateral pathway. This LTD protocol significantly depressed AMPAR EPSCs in tdT^–^ and Sal neurons, but had little impact on EPSC amplitudes of tdT^+^ neurons (Figure 7, D–F), indicating an impaired induction of LTD in these TRAPed neurons. Together, these results demonstrate that both LTP and LTD were specifically occluded postsynaptically on ELS-TRAPed tdT^+^ neurons after ELS, while no changes were seen in surrounding tdT^–^ neurons.

### Early post-ELS treatment with a specific blocker of GluA2-lacking AMPARs prevents later-life synaptic changes in ELS-TRAPed neurons.

**Figure 8:**
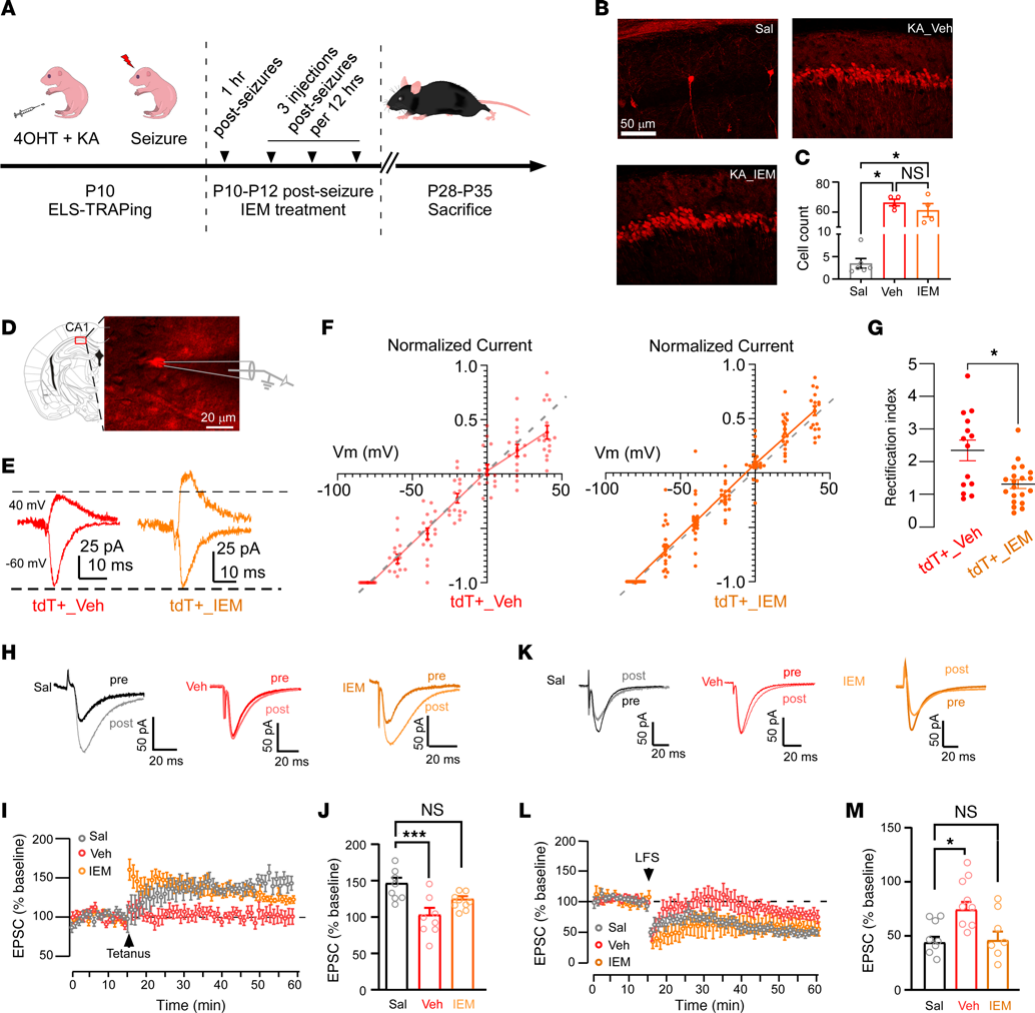
Repeated postseizure IEM-1460 treatment prevents synaptic deficits in TRAPed cells. (**A**) Experimental scheme of post-ELS IEM-1460 treatment. (**B**) Representative confocal images showing robust TRAPed cells with or without IEM-1460 treatment. (**C**) No difference in TRAPed cell count between the vehicle (Veh, *n* = 4) and IEM-1460 (*n* = 4). Sal, *n* = 6. *P < 0.05 by Kruskal-Wallis test followed by Dunn’s test. (**D**) TRAPed cells under DIC. Scale bars: 50 μm (**B**) and 20 μm (**D**). (**E**) Representative EPSC traces at –60 and +40 mV from TRAPed neurons with Veh or IEM-1460. (**F**) The I-V curves of EPSCs in tdT^+^ cells show a linear relationship in the IEM-1460 group and an inwardly rectifying relationship in the Veh group. (**G**) The rectification index was decreased in IEM-1460–treated tdT^+^ neurons (*n* = 20 neurons/11 mice) compared to Veh (*n* = 14 neurons/6 mice). *P < 0.05 by Mann-Whitney *U* test. (**H** and **I**) Example traces and plots of evoked AMPA currents before and after LTP protocol (0.3 ms, 100 Hz, separated by 20 seconds) from Sal, as well as in tdT^+^ cells with Veh and IEM-1460. Sal- and IEM-1460–treated neurons showed a long-lasting increase in EPSC amplitude, but Veh tdT^+^ neurons exhibited comparable EPSC amplitude before and after stimulation. (**J**) Summary of LTP. Sal, *n* = 8 neurons/4 mice; Veh, *n* = 8 neurons/5 mice; IEM-1460, *n* = 9 neurons/5 mice. ***P < 0.001 by 1-way ANOVA followed by Tukey’s test. (**K** and **L**) Example traces and plots of evoked AMPA currents before and after low-frequency (5 Hz, 900 pulses) LTD stimulation (LFS) from Sal, Veh, and IEM-1460 groups. Sal and IEM-1460 showed a long-lasting reduction in EPSC amplitude, whereas Veh tdT^+^ neurons exhibited comparable EPSC amplitude. (**M**) Summary of LTD. Sal, *n* = 12 neurons/6 mice; Veh, *n* = 16 neurons/6 mice; IEM-1460, *n* = 13 neurons/8 mice. *P < 0.05 by 1-way ANOVA followed by Tukey’s test. Data expressed as mean ± SEM.

IEM-1460 is a selective blocker of GluA2-lacking AMPARs, which has been shown to reduce hyperexcitability in various rodent models of epilepsy (23, 27). Moreover, previous research has demonstrated that IEM-1460 can rescue the phenotype of a mouse model of CDKL5 deficiency disorder (CDD), characterized by intellectual disability, autistic-like behaviors, and seizure behavior (26). We have shown that less specific AMPAR antagonists applied within 48 hours after ELS can attenuate seizure-induced changes in AMPAR expression and function, as well as behavioral changes and seizure susceptibility (5, 13), but it remained unclear whether these effects were specific to neurons previously activated by ELS or were rather nonspecific. To unambiguously determine whether these treatments specifically reverse changes in ELS-activated tdT^+^ neurons, we used KA to induce ELS in FosTRAP mice on P10 as described above. Mice with tonic-clonic seizures were treated acutely with IEM-1460 (10 mg/kg, i.p.) 1 hour after seizure induction and then received 3 more injections every 12 hours, with the control group receiving 0.9% saline as vehicle (Veh), and were examined at P28–P35 (Figure 8A). To assure that treatment effects were not due to a decrease in seizure burden, we applied the IEM-1460 after the seizure. Indeed, we found a comparable amount of tdT^+^ neurons in the CA1 of the Veh and IEM-1460 treatment groups (Figure 8, B and C), indicating IEM-1460 did not affect the ELS-induced activation.

We next compared the AMPAR I-V curves for tdT^+^ CA1 neurons between the IEM-1460 and Veh groups (Figure 8D). As expected, the tdT^+^ neurons from the ELS Veh-treated mice showed an inwardly rectifying, nonlinear AMPAR I-V relationship (Figure 8, E and F). However, tdT^+^ neurons in the IEM-1460–treated group displayed a linear relationship (Figure 8, E and F) and had a significantly lower rectification index than tdT^+^ neurons in the Veh control (Figure 8G), suggesting that early post-ELS treatment with IEM-1460 prevented GluA2-lacking AMPAR elevation at synapses onto ELS-TRAPed cells.

Finally, to determine whether the prevention of AMPAR changes following ELS would also rescue synaptic plasticity, we examined the effect of IEM-1460 posttreatment on subsequent LTP/ LTD using the same paring protocol or low-frequency LTD protocol, as previously described. Recordings were performed at P28–P35 in slices prepared from FosTRAP mice that experienced ELS with either IEM-1460 or Veh posttreatment, compared to Sal (no seizure) controls. Consistent with our prior findings, we observed disrupted LTP in tdT^+^ neurons in the Veh group, compared with robust LTP in the Sal (no seizure) controls (Figure 8, H–J). Remarkably, postseizure IEM-1460 treatment resulted in significantly greater LTP in tdT^+^ neurons compared with those from Veh-treated ELS mice (Figure 8, H–J), indicating that IEM-1460 treatment prevents LTP impairment after ELS. In addition to the rescue of LTP, we observed robust LTD in tdT^+^ neurons from IEM-1460–treated mice, similar to that in neurons from Sal controls, while as expected, the Veh-treated ELS group showed impaired LTD induction (Figure 8, K–M). Together, our results suggest that early post-ELS IEM-1460 treatment restores subsequent LTP and LTD impairments in ELS-TRAPed cells.

To directly test whether the IEM-1460 posttreatment paradigm also attenuated ELS-induced disruption of LTP and LTD at the network level, we used multielectrode array (MEA) extracellular field recordings in the Schaffer collateral pathway with acute brain slice preparations at P28–P35 (Supplemental Figure 5A). After a 15-minute baseline recording, theta-burst stimulation (TBS) was applied to induce LTP. In both the IEM-1460–treated group and Sal control, obvious PSC potentiation was detected, whereas there was no LTP induction in the Veh group (Supplemental Figure 5, B and C). We found that the majority of slices (10 of 15) from IEM-1460–treated mice exhibited LTP, but only a few slices (2 of 15) of the Veh group showed LTP (Supplemental Figure 5D). These results suggest that the rescue of synaptic plasticity in the tdT^+^ ELS neurons by IEM-1460 treatment is sufficient to improve plasticity at the network level.

## Discussion

This study is the first to our knowledge to reveal the evolution and persistence of structural and functional changes at the single neuron level that are induced by seizures in early life. The specific neurons activated by ELSs represent a unique seizure engram that contributes to network hyperexcitability and disrupted plasticity, but also possesses mitigable targets for therapeutic intervention. This study also highlights the heterogeneous activation of cells in distinct brain regions caused by ELSs throughout the brain. Improving our understanding of ELS-induced changes at a neuron-specific level will enable further identification of therapeutically targetable mechanisms.

Some of the most consistent clinical findings after ELSs are intellectual disability, behavioral disorders such as autism spectrum disorder, and epilepsy (13, 55, 56). Similarly, experimental models reveal that ELS induces persistent hyperexcitable networks, epileptogenesis, and behavioral disorders (2, 3, 5, 17, 43, 57). However, there is little information as to whether neuronal populations initially activated by ELS play a continued role in the subsequent pathology. A specific finding of this study is that subpopulations of neurons are selectively susceptible to ELS, and that these neurons possess long-lasting alterations in AMPAR expression due to both transcriptional and posttranslational downregulation of the GluA2 subunit. These changes in expression are associated with ELS-activated neurons displaying inwardly rectifying AMPAR EPSCs, premature synaptic unsilencing, and impaired synaptic plasticity. Thus, AMPARs represent a therapeutic target, and the present study reveals that early postseizure treatment with IEM-1460, a specific antagonist of GluA2-lacking AMPARs, results in prevention of these specific changes in the susceptible subset of ELS-activated neurons. These ELS-activated neurons may potentially serve as a seizure engram, and thus represent potential therapeutic targets to mitigate the lifelong consequences of seizures in the developing brain, for which there are no preventative treatments.

### ELSs differentially activate and alter specific neurons in CA1, with hyperexcitability persisting into later life.

Previous studies have shown that c-Fos, a marker of neuronal activation, increases markedly throughout the brain during seizure activity (58–60). In the current study, we utilized the FosTRAP transgenic mouse tool to investigate the long-term, cell-specific effects of ELS. To induce seizures, we employed the chemoconvulsant KA, which in the immature brain elicits an acute seizure episode without any subsequent occurrences of recurrent seizures or neuronal death (16, 61). This approach allowed us to track subsequent changes in neurons that had been activated by the seizure. LSFM revealed multiple regions activated by ELS, and consistent with prior studies, the hippocampus is one of the most highly activated regions in response to KA-induced seizures (36). Importantly, our results combining FosTRAP with c-Fos staining revealed that these neurons were also preferentially susceptible to activation by a subsequent seizure induced in later life. We observed that area CA1 of the hippocampus contained one of the highest densities of overlapping neurons, indicating that ELS-TRAPed cells represent a selective and persistently hyperexcitable subgroup of neurons. Our results are consistent with multiple other models that have identified hippocampal area CA1 as selectively vulnerable to seizures (62). Further, our results indicated that neurons activated by ELS are primed for hyperexcitability and seizures later in life, allowing investigation of factors that might induce this susceptibility. Indeed, this unique population may be responsible for the neural ensemble underlying epileptogenesis, as well as comorbid alterations in synaptic plasticity, and a detailed study of these neurons may yield new knowledge about the progression of pathologic changes as well as therapeutic strategies.

### ELS-activated neurons exhibit a unique deficit in GluA2 expression, with evidence for both transcriptional and posttranslational mechanisms for downregulation.

An important observation in this study was the presence of multiple mechanisms of GluA2 downregulation that was specific to the ELS-TRAPed neurons. *Gria2*, the mRNA that encodes the GluA2 protein, has recently been shown to be a critical regulator of network excitability (19). *GRIA2* human mutations have been associated with neurodevelopmental abnormalities, including intellectual disability, autism spectrum disorder, seizures, and developmental epileptic encephalopathy (63–65). Transgenic mouse models with transcriptional mutations resulting in loss of GluA2 expression and function reveal neurons with inwardly rectifying currents similar to those observed in our study and mice show behavioral deficits and seizures (63, 66, 67).

Several potential mechanisms exist at the transcriptional and epigenetic level that can result in the sustained downregulation of GluA2 (68, 69). While little is known as to how seizure activity might regulate *Gria2* expression, one potential mechanism is via a regulatory role for methylation of the *Gria2* 5′ region (70). Hypermethylation of the 5′ region of *Gria2* has been associated with increased frequency and intensity of seizures observed in epileptic rats (70). Moreover, RG108, a non-nucleoside DNA methyltransferase inhibitor, can prevent *Gria2* methylation and epileptogenic bursting (70). Given the critical roles that DNA methyl transferases play in development (71), their disruption by seizures may be involved in the long-term changes we observe here after ELS. Furthermore, it has been observed that the restrictive element-1 silencing transcription factor (REST) can exert its influence through epigenetic remodeling (72), leading to the silencing of target genes. Interestingly, studies have shown that REST may become upregulated following ELS (73, 74), which in turn results in a reduction in *Gria2* transcription (75).

The present results indicate that ELSs also induce the removal of GluA2 via activity-dependent phosphorylation of GluA2 at S880. This posttranslational modification destabilizes GluA2 within the postsynaptic density and promotes GluA2 endocytosis, resulting in GluA2-lacking AMPARs (76, 77). ELSs have been shown to increase the activity of protein kinases such as PKC (11), which mediates the phosphorylation of GluA2 at Ser880 (78, 79), consistent with our prior observations in a hypoxia-induced seizure model (11, 76, 77). While PKC activation is transient (12), it is possible that some persistently active protein kinases, such as the constitutively active PKC isoform, protein kinase Mζ (PKMζ), continually reconfigures the distribution of AMPARs (80).

### Persistent changes in AMPAR function in ELS-TRAPed neurons but not in surrounding neurons.

In our previous studies (13, 55), we demonstrated that ELS induces early changes in AMPAR function, but there had been no ability to identify activated neurons at later time points. Here, the FosTRAP mouse model enabled us to track the subset of neurons involved in ELS. Hence a potentially novel aspect of this study is the demonstration that persistent AMPAR functional changes are largely limited to ELS-activated (TRAPed) neurons within the hippocampus, suggesting a highly heterogeneous response and differential neuronal susceptibility to seizure-induced changes. Previous studies using electrophysiological recordings of in vivo and ex vivo brain slices from unanesthetized rats with spontaneous seizures (30, 31) and human tissue biopsies from adult patients with focal epilepsy (28, 29) have shown that seizures induce heterogeneous responses in distinct neuronal subpopulations around seizure onset. In the present study, a major finding was that ELS-TRAPed neurons undergo unique changes in AMPAR function that are not shared by surrounding cells. Specifically, in hippocampal CA1, only a subset of neurons expressed tdT after seizure, and only these tdT^+^ neurons exhibited increases in postsynaptic AMPAR function. These changes included enhanced AMPAR EPSC amplitude, decreased NMDA/AMPA ratio, as well as inwardly rectifying AMPAR currents, indicative of the persistent presence of GluA2-lacking AMPARs, which are consistent with our *Gria2* RNAscope and GluA2 immunohistochemistry results. Additionally, tdT^+^ cells exhibited diminished NMDAR-only silent synapses, which was not observed in surrounding tdT^–^ cells nor those from Sal control (no seizure) littermates. As ELS clinically can be associated with persistent seizure susceptibility, these results provide a potential therapeutically targetable mechanism that may underlie this persistent network hyperexcitability.

### ELSs impair synaptic plasticity via modifications in AMPARs.

Given the long-term cognitive deficits and neurodevelopmental delay that can be associated with ELS, it was important to determine whether the induced GluA2 changes impaired modes of synaptic plasticity. Hence, we studied LTP and LTD, as these forms of synaptic plasticity are widely thought to underlie learning and memory regulation. GluA2-lacking AMPARs play a crucial role in regulating various forms of synaptic plasticity, including silent synapse maturation. Silent synapses contain only NMDARs and are widespread in most brain areas until the third postnatal week (54). During this period, synapse unsilencing occurs through the recruitment of AMPARs, including GluA2-lacking AMPARs, which are abundant in the immature brain (6, 7, 19, 81, 82). We have previously shown that ELS can accelerate synapse unsilencing, leading to impaired synaptic plasticity at an early developmental stage (6, 13, 55). An important finding from this study is that NMDAR-dependent LTP and LTD are selectively occluded in ELS-TRAPed neurons, likely due to the accumulation of GluA2-lacking AMPARs at specialized synapses. As a result, the induction of LTP and LTD may be hindered, as the elevated synaptic levels of GluA2-lacking AMPARs impede the attainment of the LTP and LTD threshold for Ca^2+^ dynamics (83–85). We demonstrated a critical role for AMPAR changes in these processes with postseizure treatment with the GluA2-lacking AMPAR blocker, IEM-1460, preventing ELS-induced synaptic deficits and plasticity in ELS-TRAPed neurons. These results are consistent with our previous reports evaluating long-term behavioral outcomes (5), indicating a critical window when ELS triggers long-lasting consequences that may underlie associated deficits in learning and memory.

### A therapeutically targetable mechanism for ELS-induced neuronal dysfunction.

Pharmacological agents that broadly inhibit AMPARs have been proposed as potential antiseizure and antiepileptogenic treatments (86–89). We and others have shown that broad-spectrum AMPAR blockers, some of which are FDA approved, have efficacy both as seizure-suppressing and antiepileptogenic agents (5, 13, 90, 91). Our studies utilizing these agents have demonstrated that early postseizure treatment can effectively prevent long-lasting changes in network plasticity, seizure susceptibility, and behavioral deficits following ELS (5, 12). However, these broad AMPAR antagonists are limited by their nonspecific effects. More recently, more specific blockers of GluA2-lacking receptors have been developed, such as the adamantine derivative IEM-1460 (25), which we and others have shown to be protective in vivo in both epilepsy and stroke models (26, 27, 92–94). Our present results are the first to our knowledge to show that this agent, when administered after ELS, can prevent subsequent changes in AMPAR function in tdT^+^ neurons, specifically, without altering the ELS or initial ELS neuronal activation. These results align with our previous studies showing the therapeutic potential of IEM-1460 in seizure threshold, social interaction, and working memory deficits (26). Taken together, these data suggest that neurons activated by ELS have persistent increases in hippocampal GluA2-lacking AMPARs that contribute to hyperexcitable circuit formation and behavioral changes. These results also indicated that there may be a critical window after ELS when selective blockade of GluA2-lacking AMPARs may mitigate the persistence of the hyperexcitable network in later life.

To conclude, our study sheds light on the cellular mechanisms underlying the variable neuronal behaviors observed in the aftermath of seizures and the long-term network consequences associated with epilepsy. We found that the populations of neurons initially activated by ELS underwent persistent synaptic changes not found in surrounding neurons within the same animals, which were pharmacologically reversible and may contribute to increased seizure susceptibility and cognitive deficits later in life. Thus, our findings indicate the therapeutic promise of blocking GluA2-lacking AMPARs and that investigation of selectively activated neurons may yield novel mechanisms and therapeutic targets for seizures during neurodevelopment to better address this treatment gap.

## Methods

### Sex as a biological variable.

Both male and female mice were included in the present study. Multiple linear regression analyses were conducted to investigate the possibility of sex-related effects. No significant differences between sexes were found in all experiments of the present study. Details of statistical analyses on sex are provided in Supplemental Table 3.

Further details are provided in Supplemental Methods.

### Mice.

FosTRAP (FosCreER;Ai14) mice were randomly assigned to experimental or treatment groups and matched for age, sex, and littermate controls. All efforts were made to minimize animal suffering and numbers.

### Seizure induction and drug treatments.

ELSs were induced on P10 using KA (2 mg/kg, i.p.). Mice were given 4-OHT (50 mg/kg) 30 minutes before KA-induced tonic-clonic seizures to label ELS-TRAPed neurons. In some experiments, IEM-1460 was freshly prepared (5 mg/mL) and administrated 1 hour after ELS, followed by 3 more injections every 12 hours. A subgroup of ELS mice were subjected to KA-induced (15 mg/kg, i.p.) LLSs at P70–P100 to detect the colocalization of ELS- and LLS-associated neurons.

### Brain tissue analysis.

Mice were sacrificed at P14–P16, P28–P35, or P70–P100 for (a) immunohistochemistry, (b) LSFM, (c) RNAScope, (d) whole-cell patch-clamp recording, and (e) MEA.

### Statistics.

Details of all statistical analyses are provided in Supplemental Table 2. All statistics were calculated using GraphPad Prism 9. Data are presented as mean ± SEM. Before running the *t* test or ANOVA test, deviation from normality and the homogeneity of variance tests were evaluated by the Kolmogorov-Smirnov test and Levene’s test, respectively. A 2-tailed, unpaired or paired *t* test and 1- or 2-way ANOVA test followed by Tukey’s multiple-comparison test was applied to all data that met these 2 conditions. If either the condition of equal variances or normality was not met, statistical analyses were performed using nonparametric tests. For comparison across 2 groups, we used the Mann-Whitney *U* test for unpaired experimental design. Comparisons across more than 2 groups were implemented using Kruskal-Wallis ANOVA. Fisher’s exact test was used to determine whether there was a significant difference between 2 proportions in Supplemental Figure 5D. For experiments that include multiple data points from the same biological sample, we pooled the cell-level data from each biological replicate and compared the sample-level means. In all cases, differences were considered significant at *P* values of less than 0.05.

### Study approval.

All procedures were performed in accordance with the NIH *Guide for the Care and Use of Laboratory Animals* (National Academies Press, 2011) and with the approval of the Institution of Animal Care and Use Committee at the University of Pennsylvania.

### Data availability.

Data are available in the Supporting Data Values XLS file and from the corresponding author upon request.

## Authors contributions

FEJ conceptualized the study. BX, AJB, JV, XL, JFP, and EL developed the methodology. BX, AJB, JV, XL, SD, and EL conducted experiments. BX, AJB, and FEJ wrote the original draft of the manuscript, which was reviewed and edited by BX, AJB, JV, EL, DMT, and FEJ. FEJ acquired funding. FEJ and DMT provided resources. BX, DMT, and FEJ supervised the study.

## Supplementary Material

Supplemental data

Supplemental table 1

Supplemental table 2

Supplemental table 3

Supplemental video 1

Supporting data values

## Version 1 01/16/2024

In-Press Preview

## Version 2 03/01/2024

Electronic publication
