# Supplementary material for: Reversible synaptic adaptations in a subpopulation of murine hippocampal neurons following early-life seizures
Source: J Clin Invest. 2024 Jan 16;134(5):e175167. doi: 10.1172/JCI175167 (PMC10904056; doi:10.1172/JCI175167)
Supplement: Supplemental data [file jci-134-175167-s062.pdf]

Supplemental Methods

Animals. $Fos^{2A\text{-}iCreER}$ (TRAP2), Fos^{CreER} (TRAP1), B6.Cg-*Gt(ROSA)26Sor*$^{tm14(CAG\text{-}tdTomato)Hze}$/J(Ai14) of the congenic C57BL/6 background were obtained from The Jackson Laboratory (stock # 030323; 021882; 007914). Hemizygous offspring were generated from the original TRAP2 and TRAP1 breeders, whereby heterozygous male mice were later crossed with homozygous Ai14 female mice to obtain TRAP;Ai14 mice. Pups were weaned and rehoused on postnatal day 21. Animals were housed 2-5 per cage on a 12-hour light/dark cycle with *ad libitum* access to rodent chow and water. Both male and female mice were used for all experiments.

Drug preparation. Kainic acid (KA, Tocris), which was used to induce early-life seizures, was freshly prepared at 1 mg/mL in 0.9% sodium chloride saline solution (saline or Sal, Fisher Scientific). The preparation of 4-hydroxytamoxifen (4-OHT, Sigma) has been previously published (1). Briefly, 4-OHT was dissolved in ethanol at 20 mg/mL by shaking at 37°C for 15 min and stored at –20°C for up to several weeks. To prepare a 10 mg/mL 4-OHT working solution, Chen Oil (a 1:4 mixture of castor oil and sunflower seed oil, Sigma) was added, and the ethanol was evaporated by vacuum for 2-3 hours. The working solution was stored at 4°C for up to 36 hrs.

IEM-1460 treatment. IEM-1460 (Abcam, catalog #141507) was freshly prepared at a concentration of 5 mg/ml in saline. At P10, the FosTRAP mice with KA-induced tonic-clonic seizures received an intraperitoneal injection of IEM-1460 at 5 mg/kg 1 hr post-seizures and three more injections every 12 hrs.

Early-life seizures. Early-life seizures (ELS) were induced in FosTRAP mice at postnatal day 10 (P10). In the typical procedure, the home cage housing the P10 pups, and their dam were translocated from the housing room to a quiet procedure room for 30

min, and the pups were then separately placed in the cells of a custom-made Plexiglass chamber with 37°C heating pad. After 30 min habituation, the pups received 50 mg/kg 4-OHT (*i.p.*). After 30 min, KA (2 mg/kg) or the same amount of saline was intraperitoneally injected, and the mice were then video monitored in the chamber for 2 hrs. Videos were scored offline by a blinded observer using a modified Racine scale (2), 0, no response; 1, facial jerks, pawing; 2, nodding, wet-dog shakes, myoclonic jerks; 3, forelimb clonus; 4, loss of posture, hindlimb tonic–clonic movements; 5 status epilepticus and death. Seizure latency and duration were also recorded. Mice not reaching generalized tonic-clonic seizures were eliminated from the present study except the experiment comparing the number of TRAPed CA1 neurons between non-seizing and seizing animals (Figure S3).

Later-life seizures. Some of the mice with KA-ELS or Sal injection at P10 were subjected to later-life seizures in adulthood (2- to 3-month-old). Mice were injected with a single dose of KA (15 mg/kg, i.p.) and placed in a Plexiglas grid of 12 individual chambers. Their behavioral seizures were scored by a blinded observer using a modified Racine scale (3)(4) as follows: 0, normal behavior; 1, freezing or immobility for at least 5 s; 2, myoclonic jerks; 3, forelimb clonus; head bobbing, and scratching; 4, rearing, loss of posture, and falling; 5, generalized tonic-clonic seizure, 6, death.

Fluoro-Jade B staining. A subgroup of mice were subjected to Fluoro-Jade staining assay to confirm the established literature that KA induced seizures within the first two weeks of life do not cause neuronal death in CA1 region of hippocampus (Figure S1) (5). P11 FosTRAP mice were sacrificed by decapitation 24 hrs post 4OHT + saline/KA injections. Brains were removed and drop-fixed in 4% paraformaldehyde for 24 hrs. Following fixation, brains were dehydrated in increasing gradient of 10%, 20%, and 30% PBS-sucrose, frozen in OCT on dry ice, and stored at -80°C. Sections were cut at 20 μm

on a cryostat (CM1850, Leica Biosystems, Germany). Sections were mounted onto positively charged slides and dried overnight. Fluoro-Jade B (TR-150-FJB, Biosensis) staining was carried out according to the manufacturer's instructions. Sections were imaged acquired on a confocal microscope (Leica TCS SP8, Leica, Germany). Fluro-Jade B staining of degenerated neurons was assessed using ImageJ software (NIH). A threshold was applied to Gaussian-smoothed (sigma =1 μm, background-subtracted maximum intensity projections to create binary images for subsequent particle analysis and FJB+ cell quantification.

Immunohistochemistry, cell counting and synaptic puncta analysis. P28—P35 mice were deeply anesthetized with sodium pentobarbital (90 mg/kg; i.p.) and transcardially perfused with 4% paraformaldehyde in phosphate-buffered saline (PBS, pH 7.4). Brains were dissected and post-fixed in 4% paraformaldehyde for 24 hrs, then placed in 30% sucrose at 4 °C for cryoprotection. Brains were then sectioned coronally with a cryostat (CM1850, Leica Biosystems, Germany) at 40 μm for immunohistochemistry.

Sections were permeabilized and blocked with PBS containing 0.3% Triton X-100 (Sigma-Aldrich, MO, USA) and 5% normal goat serum (Equitech Bio, Kerrville, TX, USA). Sections were incubated overnight at 4°C with primary antibodies in the blocking solution. Primary antibodies used included: mouse anti-NeuN (1: 1,000, #MAB377, Millipore), mouse anti-GAD67 (1: 1,000, #MAB5406, Millipore), mouse anti-GFAP (1: 500, #835301, Biolegend), rabbit anti-IBA1 (1:500, #PA5-27436, Invitrogen), rabbit anti-DsRed (1:2,000, #632496, Takara Bio), rat anti-c-Fos (1:1,000, #226 017, Synaptic Systems), rabbit anti-GluA1 (1:1,000, #ab31232, abcam), rabbit anti-GluA2 (phospho S880) (1:2,000, #ab52180, abcam), rabbit anti-GluA2 (1:1,000, #182 103, Synaptic Systems), rabbit anti-Synapsin (1:1,000, #AB1543, Millipore), and chicken anti-MAP2

(1:1,000, ab5392, abcam). Sections were subsequently incubated for 2 hrs at room temperature with secondary antibodies [1:1000, Alexa Fluor 488, 593, or 647 goat anti-mouse (A-11001, A-11005, A-21235), anti-rabbit (A-11008, A-11012, A-21245), anti-rat (A-11006), or anti-chicken (A11042) antibodies, Invitrogen], transferred to Superfrost Plus slides and cover slipped using DAPI Fluoromount-G mounting media (SouthernBiotech). Images of immunostainings were acquired on a confocal microscope (SP5 and SP8, Leica, Germany) using a 20x and 63x objective. The tdT+ and immunopositive cells were manually quantified by a blind experimenter using Image J. For synaptic GluA1/GluA2 immunohistochemistry and image analysis, to prevent thresholding artifacts, colocalization was initially assessed at the maximum threshold, encompassing all GluA2 puncta. Subsequently, the GluA2 channel underwent thresholding at various intervals of the maximum threshold (95%, 90%, 85%, 80%, and 75%), with colocalization re-evaluated at each threshold. Following channel thresholding, the identification of synapsin and GluA1/GluA2 puncta was conducted utilizing the FIJI "Coloc 2" function. The region of interest (ROI) was manually defined over the CA1 region of the hippocampus.

RNAScope. For *Gria1* and *Gria2* mRNA detection by RNAscope, FosTRAP mice at P32—P35 were perfused as described in the ‘**Immunohistochemistry’** section. The fixed brains were cryoprotected in graded sucrose solutions (10%, 20%, and 30% sucrose in PBS) for 24—48 hrs each, sectioned (20 μm) using a cryostat (CM1850, Leica Biosystems, Germany), and stored at −80°C until used. For RNAscope staining, the sections were mounted on Superfrost Plus slides, dried in an oven for 30 min at 40°C, and then RNAScope^{TM} Multiplex Fluorescent Reagent Kit V2 (catalog #323100, Advanced Cell Diagnostics) was applied over the sections according to the manufacturer’s instructions. *Gria1* probe (RNAscope Probe- Mm-*Gria1*-C2; catalog

#426241, Advanced Cell Diagnostics) and *Gria2* probe (RNAscope Probe- Mm-*Gria2*-C3; catalog #416091, Advanced Cell Diagnostics) for RNAscope Multiplex Fluorescent assay was designed and synthesized by Advanced Cell Diagnostics. After hybridization, sections were immunostained with NeuN. Images of immunostainings were acquired on a confocal microscope (Leica TCS SP8) using a 63x objective. The fluorescent intensities of *Gria1* and *Gria2* in tdT+/NeuN+ and tdT-/NeuN+ in the pyramidal cell layer of CA1 were quantified using Image J.

Brain clearing. FosTRAP mice were transcardially perfused with 4% paraformaldehyde and incubated in 4% paraformaldehyde at 4° C overnight. Fixed brains were then hemisected and cleared following a modified CUBIC brain-clearing protocol (6, 7). Briefly, intact hemispheres underwent incubations with slow rotation at 37°C sequentially with overnight incubation in 1:1 CUBIC-L (TCI America, Portland, OR, USA): water, 24 hrs 100% CUBIC-L, and 48 hrs 100% CUBIC-L. Brains were then washed 3 x 1hr in PBS and stored at 4° C for up to one week. Delipidated hemispheres then underwent refractive index homogenization by incubation at 37° C with slow rotation in 1:1 CUBIC Reagent-2 (made in-lab, (7)) : PBS for 6 hrs and then 100% CUBIC Reagent-2 overnight. CUBIC Reagent-2 was replaced, and brains were imaged 24 hrs later.

Light sheet fluorescence microscopy (LSFM). Cleared, intact FosTRAP mouse hemispheres were adhered by the brainstem to a custom adapter and submerged in CUBIC reagent-2 in the imaging chamber. LSFM was performed with Zeiss Lightsheet Z.1 (Zeiss, Oberkochen, DE) with sCMOS pco. edge camera (PCO Tech, Wilmington, DE, USA) and 5x/0.16 NA imaging objective with dual side 561 and 488 nm excitation lasers with 585 nm long pass and 550 nm lowpass filters for tdTomato and autofluorescence illumination, respectively. Tiles were imaged with 4.73 μm Z-step

through the sagittal plane at 0.8X zoom with 1.15 x 1.15 μm pixel dimensions. Tiles were acquired with a 5% overlap.

Brain mapping. Tiled images were stitched in Arivis Vision 4D (Zeiss) and the autofluorescence channel was exported as a tiff stack. Linear Allen Brain Atlas (ABA) registration was performed with QuickNII (8). The resulting ABA image stack was converted to 32-bit greyscale and binned every 50-200 images based on anatomical landmarks. To refine ABA alignment to corresponding autofluorescent images, further nonlinear (thin plate spline) refinement was performed to each bin by landmark-based transformations with the Big Warp plugin for Image J (9). Detection of tdT+ cells was performed with Blob Finder in Arvis Vision 4D. Registered, 32-bit greyscale ABA images were imported into Arivis and cell detection results were applied to the atlas. 32-bit intensity values for each cell were exported and matched to the corresponding brain region with custom code in R.

Brain slice preparation. Mice were anesthetized with sodium pentobarbital (90 mg/kg; i.p.) and perfused with ice-cold, oxygenated artificial cerebrospinal fluid [aCSF, (in mM) 124 NaCl, 2.5 KCl, 1.2 NaH_2PO4, 24 $NaHCO_3$, 5 4-(2-hydroxyethyl)-1-piperazineethanesulfonic acid (HEPES), 12.5 glucose, 2 $CaCl_2 \cdot 2H2O$, and 2 $MgSO_4 \cdot 7H_2O$]. The brains were rapidly removed, and horizontal hippocampal slices were cut (300 μm) (10) using a Leica VT1000s vibratome in an ice-cold bath of oxygenated aCSF. Slices recovered for 15 min at 34 °C in an NMDG-aCSF solution (in mM, 93 N-methyl-D-glucamine (NMDG), 2.5 KCl, 1.2 NaH_2PO_4, 30 $NaHCO_3$, 20 HEPES, 25 glucose, 2 thiourea, 5 sodium ascorbate, 3 sodium pyruvate, 0.5 $CaCl_2 \cdot 2H_2O$ and 10 $MgSO_4 \cdot 7H_2O$, pH 7.3–7.4), then for ≥ 1 hr at room temperature in oxygenated aCSF until used for recording.

Whole-cell patch-clamp recording. Brain slices were placed into a recording chamber mounted on a Nikon upright microscope (E600FN) equipped with IR-DIC and fluorescence optics, where they were bathed at 30–32°C in oxygenated aCSF (bath flow at ~2 mL/min). All recordings were made in the CA1 region of hippocampus. The ELS-TRAPed neurons were identified by the presence of tdTomato fluorescence. Somatic whole-cell voltage-clamp recordings were obtained from pyramidal cells using patch electrodes with an open tip resistance of 2–5 MΩ. Pipettes were filled with an internal solution composed of, in mM: 110 Cs methanesulfonate, 10 TEA-Cl, 4 NaCl, 2 $MgCl_2$, 0.5 EGTA, 10 HEPES, 4 ATP-Mg, 0.3 GTP, 7 phosphocreatine, creatine phosphokinase (17 units/mL), 1 QX-314, and 0.1 spermine, pH 7.3–7.4, 270–280 mOsm, except for recording of intrinsic properties, which was, in mM: 126 K-gluconate, 4 KCl, 0.3 EGTA, 10 HEPES, 4 ATP-Mg, 0.3 GTP, 10 phosphocreatine, pH 7.3, mOsm 270-280) (11, 12). Whole-cell patch-clamp recordings were performed using Multiclamp 700B amplifier and Clampex 11 software (Molecular Devices). Data were filtered at 2 kHz and digitized at 20 kHz using a Digidata 1550B analog-to-digital converter (Molecular Devices). The membrane potentials were not corrected for liquid junction potential.

For recordings of AMPAR-mediated spontaneous EPSCs (sEPSCs), cells were voltage-clamped at −60 mV and recorded for 5–10 min in the presence of 60 μM picrotoxin (Tocris) and 50 μM D-AP5 (Abcam). All data were analyzed offline using Clampfit 10.2 (Molecular Devices). A typical EPSC was selected to create a sample template for the event detections within a 5–10 min data period. The sEPSC events were detected automatically with a threshold of 5–6 pA (2 times the root mean square of the noise). The frequency (number of events) and amplitude of the individual events were examined with the threshold set at the medium level (i.e., 5) in Clampfit. The detected events were then visually confirmed. The weighted decay time constant (τ_{dw}) was calculated by first

fitting a double-exponential function on the averaged sEPSC trace for each neuron that excluded multiple overlapping sEPSC events: $EPSC(t) = w_1 \exp(-t/\tau_1) + w_2 \exp(-t/\tau_2)$ and then using the fit values in the following equation: $\tau_{dw} = (w_1 \tau_1 + w_2 \tau_2)/(w_1 + w_2)$ (13, 14).

For recordings of electrically evoked EPSCs (eEPSCs), synaptic currents were elicited by stimuli (10 s intervals, 0.1 ms duration, 40-60% maximum response) through a tungsten concentric bipolar microelectrode (inner diameter: 3–4 μm; outer diameter: 127 μm, World Precision Instruments) placed in the Schaffer collaterals, 150–250 μm from the soma of the recorded cell. To examine AMPAR subunit composition, evoked EPSCs were measured at the membrane potentials from -80 mV to 40 mV with 20 mV steps, and the I-V curve was plotted from the average peak amplitude from 3 or 4 recordings per holding voltage. The rectification index was calculated as the peak amplitude of $AMPA-EPSC_{-60\ mV}/AMPA-EPSC_{40\ mV}$ as reported (12). In some experiments, relative changes to baseline $AMPA\text{-}EPSC_{-60mV}$ amplitude (onset to peak) were measured following a 5-min bath application of IEM-1460 (100 μM). To measure the synaptic strength, AMPAR-eEPSCs were recorded at a holding potential of −60 mV in the presence of picrotoxin (60 μM), then the AMPAR antagonist NBQX (20 μM) was bath applied at a holding potential of +40mV to isolate NMDAR-EPSCs. The AMPAR/NMDAR ratio was calculated by dividing the peak amplitudes.

Silent synapses were assessed using the minimal stimulation assay as described previously (11, 15). In the presence of picrotoxin (60 μM), AMPAR-eEPSCs or NMDA-eEPSCs were recorded at a holding potential of −60 mV or +40 mV, respectively. After obtaining small (~50 pA) AMPAR-eEPSCs at −60 mV, the stimulation intensity was adjusted until failure responses of 50–60% at −60 mV to consecutive 60–200 trials were reached (16, 17). This final stimulus intensity was then kept constant to evoke NMDAR-eEPSCs at +40 mV for the same cells. All failure responses were visually checked and

verified. Corresponding eEPSC failure rates at −60 and +40 mV and fraction of silent synapses [1-ln($F_{-60\ mV}$)/ln($F_{+40\ mV}$)] were calculated using published methods (11, 16).

For eEPSC-LTP and eEPSC-LTD studies, AMPAR-eEPSCs were evoked (0.05 Hz, 0.1 ms duration) at Schaffer collaterals and the stimulation intensity was adjusted to evoke synaptic currents ~50–60% maximal response (range; 50–250 µA) (11, 15). A stable baseline was recorded for 15 min, cells were held at +10 mV with 2 tetani (0.3 ms, 100 Hz, separated at 20 s) to induce activity-dependent LTP, or cells were held at -40 mV with 900 pulses at 5 Hz [long frequency stimulation (LFS), 0.3 ms duration] to induce NMDAR-dependent LTD, followed by a post-tetani or post-LFS recording period of at least 45 min. The magnitude of LTP or LTD was determined by comparing average EPSCs recorded 35–45 min after induction to EPSCs recorded 5–15 min before induction. Series resistance was monitored continuously and experiments showing a greater than 20% change were excluded.

Multielectrode array recordings (MEA). Mice were euthanized, brains were dissected, and acute slices were generated as described in the '**Brain slice preparation'** section. The slice was placed over an 8 × 8 array of planar electrodes, each 20 × 20 µm in size, with an interpolar distance of 100 µm (MED-P2105, Alpha MED Sciences). During recording, the slice was kept submerged in oxygenated aCSF at 30–32 °C using a platinum ring covered with nylon mesh. Voltage signals were recorded with the MED64 System (Alpha MED Sciences) and digitized at 20 kHz followed by filtering at 0.1–1 Hz with a 6071E Data Acquisition Card (National Instruments), using Mobius software (Alpha MED Sciences) as our previous study (13). The field excitatory postsynaptic potentials (fEPSP) were evoked by stimulating one of the 64 planar electrodes placed in the Schaffer collateral pathway and recorded in the CA1 area of the hippocampus. The recording electrode with the highest fEPSP amplitude, at a distance of at least 100 µm

from the stimulation site, was selected as the recording site. After a stable baseline was established for 5 min, the Input-output (I-O) curves were obtained by measuring the fEPSP initial slope at increasing 5 μA steps of afferent stimulation, at a 30 s-interval. For LTP experiments, the stimulation intensity was adjusted to evoke synaptic currents ~at 50% of maximal amplitude (range; 75–300 μA). After at least 15 min baseline recording, attempts to induce LTP at the Schaffer collateral pathway were made by delivering theta-burst stimulation (3 bursts with a 20 s interburst interval, each burst consisted of 4 pulses with a duration of 0.2 ms at a frequency of 100 Hz), followed by post-HFS LFP recording for at least 45 min. The degree of LTP was evaluated by the fEPSP average amplitude at 40–45 min after the theta-burst stimulation, normalized to the average amplitude during baseline.

References

1. Xing B, et al. A Subpopulation of Prefrontal Cortical Neurons Is Required for Social Memory. *Biol Psychiatry.* 2021;89(5):521-531.
2. Rakhade SN, et al. Glutamate receptor 1 phosphorylation at serine 831 and 845 modulates seizure susceptibility and hippocampal hyperexcitability after early life seizures. *J Neurosci.* 2012;32(49):17800-17812.
3. Luttjohann A, et al. A revised Racine's scale for PTZ-induced seizures in rats. *Physiol Behav.* 2009;98(5):579-586.
4. Koh S, et al. NBQX or topiramate treatment after perinatal hypoxia-induced seizures prevents later increases in seizure-induced neuronal injury. *Epilepsia.* 2004;45(6):569-575.
5. Schmued LC, et al. Fluoro-Jade C results in ultra high resolution and contrast labeling of degenerating neurons. *Brain Res.* 2005;1035(1):24-31.

6. Matsumoto K, et al. Advanced CUBIC tissue clearing for whole-organ cell profiling. *Nat Protoc.* 2019;14(12):3506-3537.
7. Susaki EA, et al. Whole-brain imaging with single-cell resolution using chemical cocktails and computational analysis. *Cell.* 2014;157(3):726-739.
8. Puchades MA, et al. Spatial registration of serial microscopic brain images to three-dimensional reference atlases with the QuickNII tool. *PLoS One.* 2019;14(5):e0216796.
9. Bogovic JA, et al. *2016 IEEE 13th international symposium on biomedical imaging (ISBI)*. IEEE; 2016:1123-1126.
10. Bischofberger J, et al. Patch-clamp recording from mossy fiber terminals in hippocampal slices. *Nat Protoc.* 2006;1(4):2075-2081.
11. Zhou C, et al. Hypoxia-induced neonatal seizures diminish silent synapses and long-term potentiation in hippocampal CA1 neurons. *J Neurosci.* 2011;31(50):18211-18222.
12. Yennawar M, et al. AMPA Receptor Dysregulation and Therapeutic Interventions in a Mouse Model of CDKL5 Deficiency Disorder. *J Neurosci.* 2019;39(24):4814-4828.
13. Song YJ, et al. Dysregulation of GABAA Receptor-Mediated Neurotransmission during the Auditory Cortex Critical Period in the Fragile X Syndrome Mouse Model. *Cereb Cortex.* 2021;32(1):197-215.
14. Xing B, et al. Juvenile treatment with mGluR2/3 agonist prevents schizophrenia-like phenotypes in adult by acting through GSK3beta. *Neuropharmacology.* 2018;137:359-371.
15. Sun H, et al. Early Seizures Prematurely Unsilence Auditory Synapses to Disrupt Thalamocortical Critical Period Plasticity. *Cell Rep.* 2018;23(9):2533-2540.

16. Isaac JT, et al. Evidence for silent synapses: implications for the expression of LTP. *Neuron.* 1995;15(2):427-434.

17. Liao D, et al. Activation of postsynaptically silent synapses during pairing-induced LTP in CA1 region of hippocampal slice. *Nature.* 1995;375(6530):400-404.

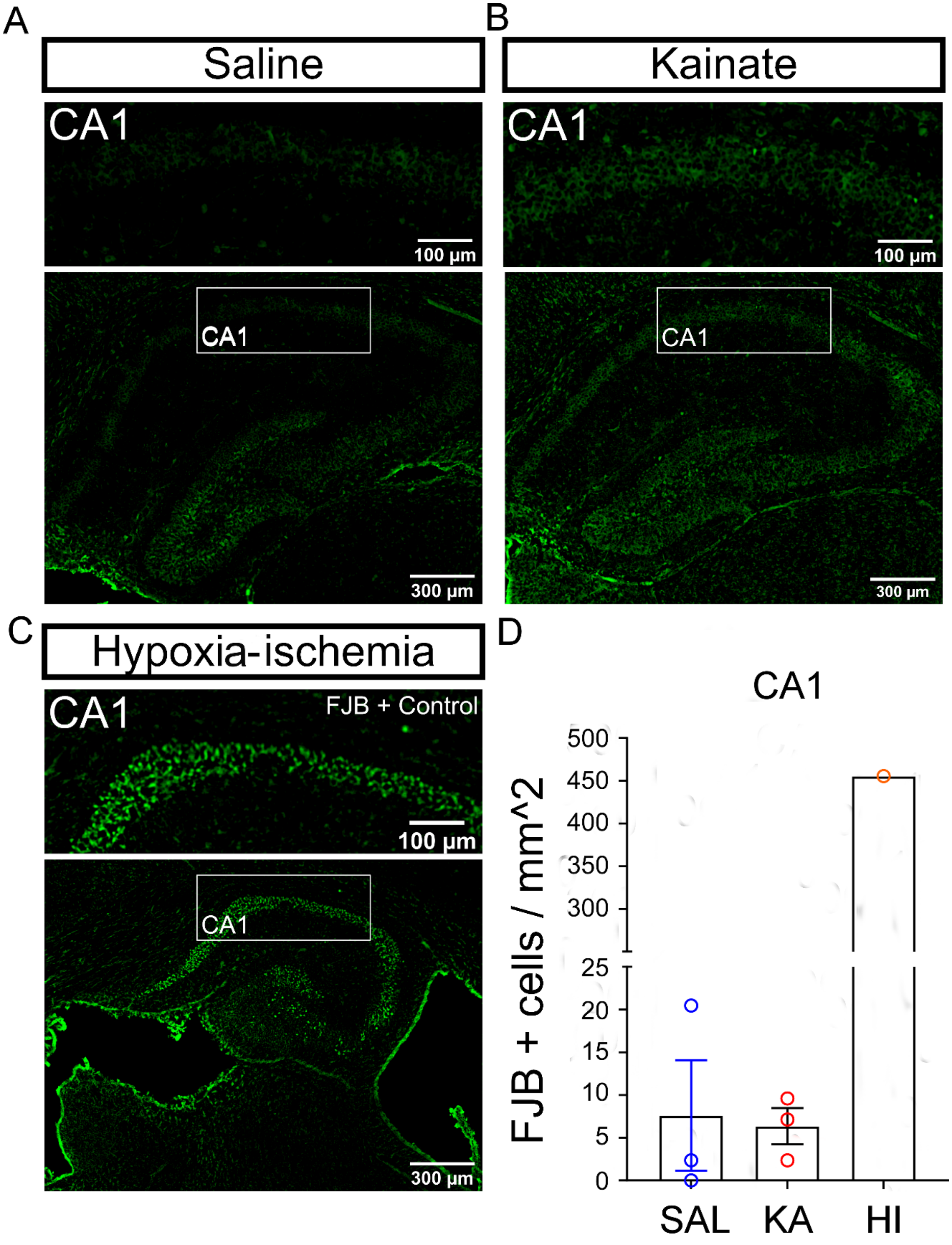

Figure S1. P10 KA-induced seizures do not produce appreciable cell death 24 hrs post KA-seizure. Representative images of Fluoro-Jade B (FJB) staining of the CA1 region (upper panels) and entire hippocampus (lower panels) from a saline control (**A**) and a mouse subjected to KA-

ELS at P10 (**B**) showing few FJB+ cells in CA1 region at P11 in either saline or KA-ELS group. (**C**) In the P9 hypoxia-ischemia (HI) neonate mouse model, the entire hippocampus, especially the CA1 region (upper panel), shows a clear pattern of neurodegeneration at P12. (**D**) Quantification of FJB+ cell density in CA1 of saline (SAL), KA and HI mice. Compared with the HI positive control (455.5 cells/mm^2, n =1), few FJB+ cells were found in either SAL (7.6 ± 7.9 cells/mm^2, n = 3) or KA (6.4 ± 4.1 cells/mm^2, n = 3) mice. Data expressed as mean ± SEM.

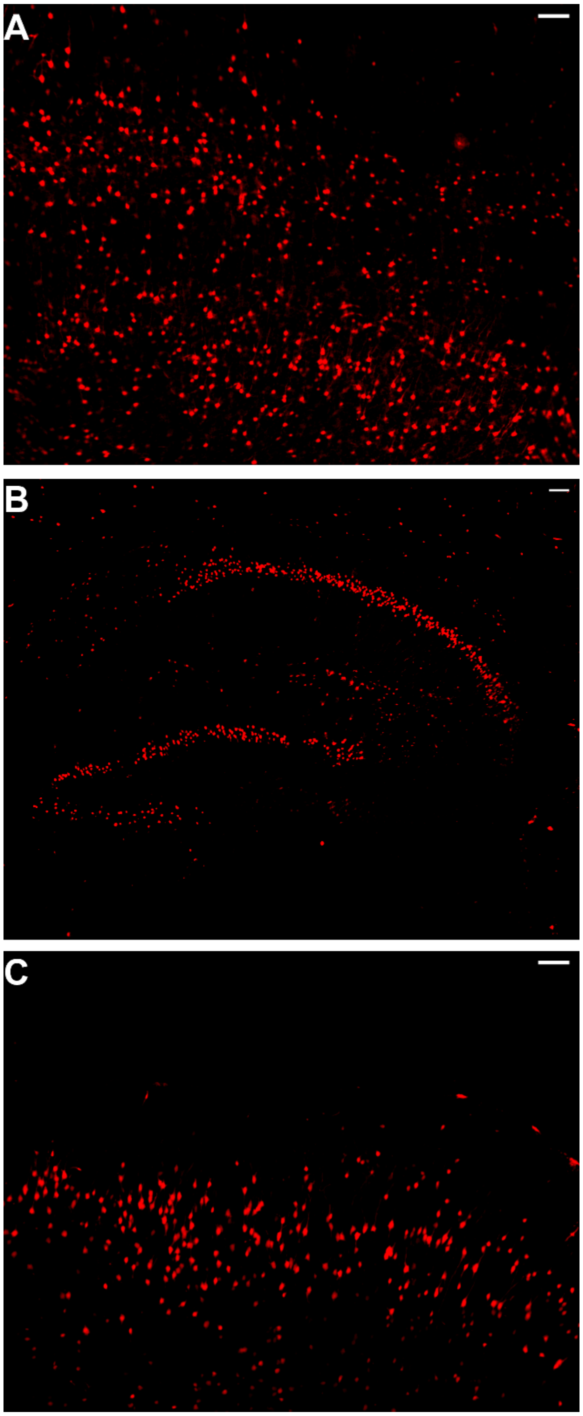

Figure S2. Representative 11-plane maximum intensity projection (~50 µm) from the (**A**) isocortex, (**B**) hippocampus, and (**C**) piriform cortex (olfactory area) from an ELS-TRAP mouse that was imaged by LSFM. Scale bars = 100 µm.

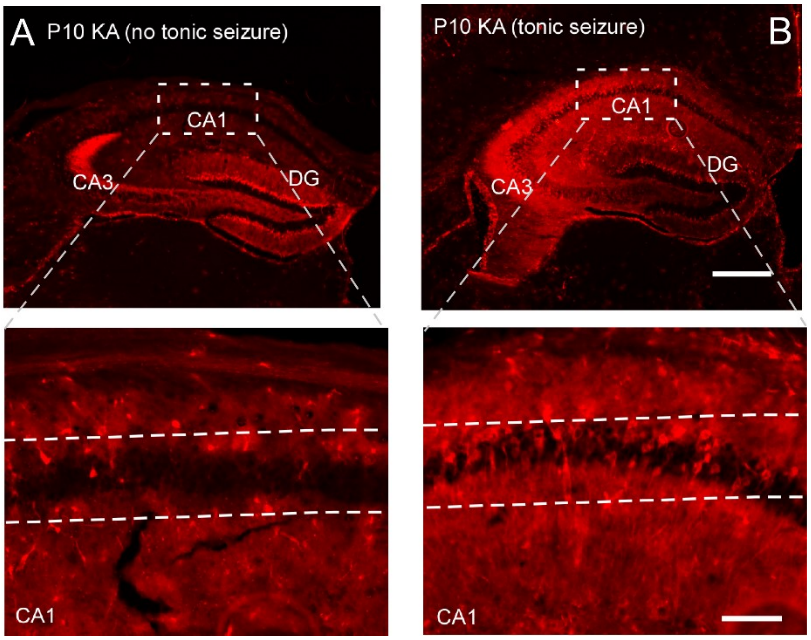

Figure S3. Representative coronal sections from FosTRAP mice injected with KA at P10. (**A**) Coronal section from a mouse that received KA but did not exhibit tonic-clonic seizures showing a limited number of TRAPed cells in the CA1 region. (**B**) Coronal section from a mouse experiencing tonic-clonic seizures, demonstrating a substantial presence of TRAPed cells in CA1. Scale bars: Upper panel, 500 μm; Lower panel, 100 μm.

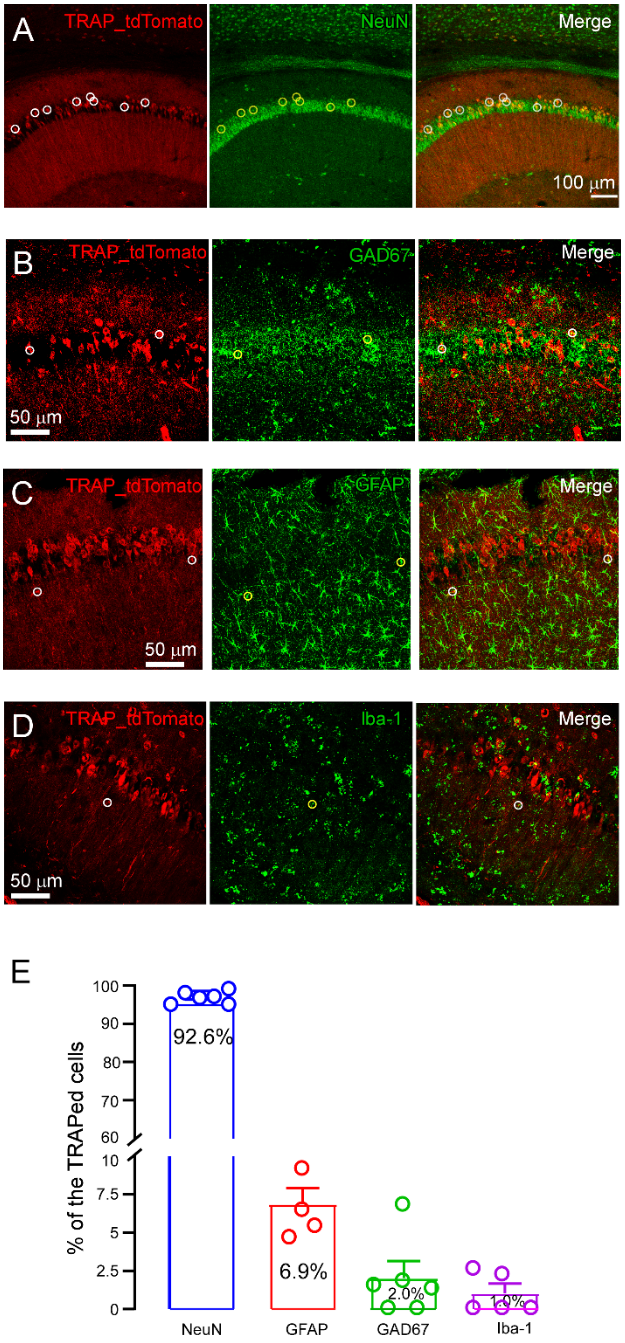

Figure S4. ELS-TRAPed cells in the CA1 region are primarily pyramidal neurons. (A-D) Representative confocal images of CA1 from ELS-TRAPed mice sacrificed at P30, co-immunostained with tdTomato and different cellular markers: **(A)** NeuN (shown again from Figure 1G, n = 6), **(B)** GAD67 for interneurons (n = 4), (**C**) GFAP for astrocytes (n = 6) and (**D**) Iba-1 (n =

5) for microglia. Circles in A-D indicate co-localization. (**E**) Quantification of TRAPed tdTomato positive cells co-labeled with each cellular marker revealed that the majority were NeuN positive. Data expressed as mean ± SEM.

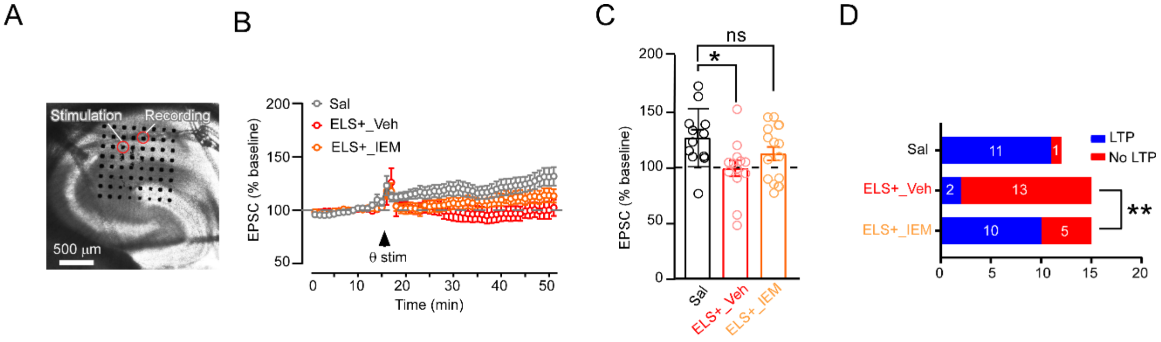

Figure S5. Post-seizure IEM treatment rescues ELS-induced CA1-CA3 LTP impairment at P28–35. (**A**) Representative image showing MEA overlaid on the hippocampal slice. (**B**) Summary plots of evoked fEPSP amplitudes before and after theta bursts LTP stimulation (θ stim) from Sal (n = 12 slices/4 mice), ELS+_Veh (n = 15 slices/6 mice), and ELS+_IEM-1460 (n = 15 slices/8 mice) treatment groups. (**C**) Quantification of LTP experiments. IEM-1460 treatment prevents ELS-induced LTP impairment. One-way ANOVA followed by Tukey's test, *$P < 0.05$. (**D**) A higher proportion of slices with LTP in the ELS+_IEM-1460 treatment compared to the ELS+_Veh group. Fisher's exact test, **$P < 0.01$.
