# Supplementary material for: Reversible synaptic adaptations in a subpopulation of murine hippocampal neurons following early-life seizures
Source: J Clin Invest. 2024 Jan 16;134(5):e175167. doi: 10.1172/JCI175167 (PMC10904056; doi:10.1172/JCI175167)
Supplement: Supplemental table 1 [file jci-134-175167-s063.pdf]

Region	Treatment	Mean tdT+ cell count	SEM
Isocortex	Saline	20630	3660.41423
	KA	103667.25	32413.95154
Olfactory Areas	Saline	9277.5	4527.5
	KA	52817	22550.00047
Hippocapal formation	Saline	1375.666667	543.8799908
	KA	90943	15419.69206
Cortical subplate	Saline	2013.666667	203.5291407
	KA	39085.25	16630.80606
Striatum	Saline	2994.333333	1104.20776
	KA	17110.5	4880.734687
Pallidum	Saline	1670.666667	1126.272663
	KA	2556	970.9527108
Thalamus	Saline	2071	1536.97571
	KA	3049.5	957.6755279
Hypothalamus	Saline	5241	4689.006967
	KA	7197	3510.290515
Midbrain	Saline	1356	916.2184965
	KA	2156.75	739.9368639
Pons	Saline	3644.666667	3127.814697
	KA	5224.5	3302.255807
Medulla	Saline	3948.666667	2770.852717
	KA	5117	2519.074069
Cerebellum	Saline	2331	1993.502696
	KA	19862.5	8688.533713

Table S1. Table of mean cell count quantifications and SEM from LSFM of Saline and ELS-TRAP mice. N=3 (Sal), 4 (KA).
