# Supplementary material for: Reversible synaptic adaptations in a subpopulation of murine hippocampal neurons following early-life seizures
Source: J Clin Invest. 2024 Jan 16;134(5):e175167. doi: 10.1172/JCI175167 (PMC10904056; doi:10.1172/JCI175167)
Supplement: Supplemental table 2 [file jci-134-175167-s064.pdf]

Data	Response variable	Groups	n define as	slice/animal used	Normality Test (Kolmogorov-Smirnov test)	Homogeneity of variance test (F test)	Hypothesis test	Test value	*P* -value	summary
Figure 1F	tdT+ cell number in dorsal→ventral axis	KA	mouse	n=4 mice			Simple linear regression	F=10.43	*P* =0.0121	*
	tdT+ cell number in medial→lateral axis			n=4 mice			Simple linear regression	F=13.98	*P* =0.0053	**
Figure 1H	tdTom+ cell density	Sal	mouse	n=6 mice	KS=0.1987; p=0.6457	F=0.0135 p=0.0002*	Mann Whitney test	U=0	*P* =0.0002	***
		KA	mouse	n=11 mice	KS=1910; p=0.3170					
Figure 1I	seizure duration with tdT+ cell density	KA	mouse	n=10 mice			Simple linear regression	F=14.34	*P* =0.0053	**
Figure 2C	Latency to KA-LLS	Sal (con)-KA (LLS)	mouse	n=6 mice	KS=0.2413; p=0.3348	F=1.8531 p=0.4740	Mann Whitney test	U=4	*P* =0.0140	*
		KA (ELS)-KA (LLS)		n=7 mice	KS=0.4022; p=0.0011*					
Figure 2D	Cumulative seizure scores	Sal (con)-KA (LLS)	mouse	n=6 mice	KS=0.3436; p=0.0257*	F=0.3488 p=0.2678	Mann Whitney test	U=5	*P* =0.0216	*
		KA (ELS)-KA (LLS)		n=7 mice	KS=0.3802; p=0.0028*					
Figure 2G	proportion among c-Fos+ cells	tdT-	mouse	n=6 mice	KS=0.1875; p=0.7313	F=10.0201 p=0.0244*	Mann Whitney test	U=0	*P* =0.0022	**
		tdT+		n=6 mice	KS=0.1760; p=0.8108					
Figure 3D	sEPSC frequency (Hz)	Sal	cell	n=11/6 mice	KS=0.2147; p=0.1676	F=3.9756 p=0.0278*	Kruskal-Wallis ANOVA	H=4.003	*P* =0.1352	ns
		tdT-		n=16/8 mice	KS=0.2627; p=0.0043*					
		tdT+		n=14/8 mice	KS=0.2810; p=0.0153*					
Figure 3E	sEPSC Amplitude (pA)	Sal	cell	n=11/6 mice	KS=0.1780; p=0.4264	F=3.5761 p=0.0832	one-way ANOVA	F=6.175	*P* =0.0049	**
		tdT-		n=16/8 mice	KS=0.1613; p=0.3671					
		tdT+		n=14/8 mice	KS=0.1949; p=0.1587					
							Tukey's multiple comparisons test	Sal vs tdT-	*P* =0.7489	ns
								Sal vs tdT+	*P* =0.0250	*
								tdT- vs tdT+	*P* =0.0069	**
Figure 3G	sEPSC frequency (Hz)	Sal	cell	n=22/8 mice	KS=0.2041; p=0.0177*	F=4.7485 p=0.0126*	Kruskal-Wallis ANOVA	H=0.8810	*P* =0.6438	ns
		tdT-		n=20/8 mice	KS=0.2230; p=0.0103*					
		tdT+		n=15/8 mice	KS=0.2275; p=0.0355*					
Figure 3H	sEPSC Amplitude (pA)	Sal	cell	n=22/8 mice	KS=0.1785; p=0.0663	F=3.2690 p=0.0457*	Kruskal-Wallis ANOVA	H=9.1750	*P* =0.0102	*
		tdT-		n=20/8 mice	KS=0.2746; p=0.0004*					
		tdT+		n=15/8 mice	KS=0.1749; p=0.2492					
							Dunn's multiple comparisons test	Sal vs tdT-	*P* >0.9999	ns
								Sal vs tdT+	*P* =0.0363	*
								tdT- vs tdT+	*P* =0.0135	*

Figure 3K	Rectification index (P14-16)	Sal	cell	n=14/5 mice	KS=1.7774; p=0.4112	F=2.8134 p=0.0807	one-way ANOVA	F=15.07	*P* <0.0001	****
		tdT-		n=13/4 mice	KS=0.2008; p=0.3695					
		tdT+		n=14/4 mice	KS=0.1994; p=0.4586					
							Tukey's multiple comparisons test	Sal vs tdT-	*P* =0.6492	ns
								Sal vs tdT+	*P* =0.0007	***
								tdT- vs tdT+	*P* <0.0001	****
	Rectification index (P28-35)	Sal	cell	n=14/5 mice	KS=0.1566; p=0.5737	F=3.2309 p=0.0512	one-way ANOVA	F=10.78	*P* =0.0002	***
		tdT-		n=13/4 mice	KS=0.1799; p=0.2978					
		tdT+		n=14/4 mice	KS=0.2170; p=0.0728					
							Tukey's multiple comparisons test	Sal vs tdT-	*P* =0.7091	ns
								Sal vs tdT+	*P* =0.0003	***
								tdT- vs tdT+	*P* =0.0034	**
Figure 3M	NMDA/AMPA ratio	Sal	cell	n=11/4 mice	KS=0.2149; p=0.1665	F=0.6035 p=0.5547	one-way ANOVA	F=9.755	*P* =0.0007	***
		tdT-		n=8/4 mice	KS=0.2308; p=0.1806					
		tdT+		n=14/4 mice	KS=0.2007; p=0.4474					
							Tukey's multiple comparisons test	Sal vs tdT-	*P* =0.1432	ns
								Sal vs tdT+	*P* =0.0310	*
								tdT- vs tdT+	*P* =0.0005	***
Figure 4B	Gria2/Gria1 ratio	tdT-	cell	n=148/3 mice	KS=0.1910; p=1.265E-9*	F=1.2736 p=0.1540	Paired t test	$t_{(2)}$=4.710	*P* =0.042	*
		tdT+	cell	n=136/3 mice	KS=0.1152; p=0.0023*					
Figure 5C	GluA1/Synapsin colocalization	Sal	mouse	n=7 mice			RM two-way ANOVA	F interaction=0.0033	*P* >0.9999	ns
		tdT-		n=6 mice				F threshold=2.515	*P* =0.0348	*
		tdT+		n=6 mice				F group=2.425	*P* =0.0938	ns
Figure 5F	GluA2/Synapsin colocalization	Sal	mouse	n=7 mice			RM two-way ANOVA	F interaction=0.0501	*P* >0.9999	ns
		tdT-		n=6 mice				F threshold=6.649	*P* <0.0001	****
		tdT+		n=6 mice				F group=2.425	*P* <0.0001	****
							Tukey's multiple comparisons test	100% Sal vs tdT-	q=1.354, df=96	ns
								100% Sal vs tdT+	q=5.867, df=96	***
								100% tdT- vs tdT+	q=4.349, df=96	**
								95% Sal vs tdT-	q=1.317, df=96	ns
								95% Sal vs tdT+	q=5.543, df=96	***
								95% tdT- vs tdT+	q=4.072, df=96	*
								90% Sal vs tdT-	q=1.258, df=96	ns
								90% Sal vs tdT+	q=5.259, df=96	***
								90% tdT- vs tdT+	q=3.856, df=96	*
								85% Sal vs tdT-	q=1.116, df=96	ns
								85% Sal vs tdT+	q=4.957, df=96	**
								85% tdT- vs tdT+	q=3.702, df=96	*
								80% Sal vs tdT-	q=1.079, df=96	ns
								80% Sal vs tdT+	q=4.825, df=96	**
								80% tdT- vs tdT+	q=3.609, df=96	*
								75% Sal vs tdT-	q=1.084, df=96	ns
								75% Sal vs tdT+	q=4.765, df=96	**
								75% tdT- vs tdT+	q=3.547, df=96	*

Figure 5K	pGluA2 intensity (AU)	Sal	cell	n=100/5 mice	KS=0.0735; p=0.2035	F=21.5926 p=1.7879E-9*	One-way ANOVA	F=3.970	*P* = 0.0353	*
		tdT-		n=96/9 mice	KS=0.1555; p=5.423E-6*					
		tdT+		n=100/9 mice	KS=0.1109; p=0.0041*					
							Tukey's multiple comparisons test	Sal vs tdT-	*P* =0.6862	ns
								Sal vs tdT+	*P* =0.0435	*
								tdT- vs tdT+	*P* =0.1181	ns
Figure 6D	proportion of silent synapses	Sal	cell	n=7/3 mice	KS=0.1735; p=0.7560	F=6.2081 p=0.0101*	Kruskal-Wallis ANOVA	H=11.79	*P* =0.0004	***
		tdT-		n=6/3 mice	KS=0.2183; p=0.4946					
		tdT+		n=6/3 mice	KS=0.1906; p=0.7078					
							Dunn's multiple comparisons test	Sal vs tdT-	*P* >0.9999	ns
								Sal vs tdT+	*P* =0.0046	**
								tdT- vs tdT+	*P* =0.0168	*
Figure 6H	proportion of silent synapses	Sal	cell	n=9/3 mice	KS=0.2480; p=0.7560	F=3.8005 p=0.0375*	Kruskal-Wallis ANOVA	H=3.604	*P* =0.1649	ns
		tdT-		n=9/3 mice	KS=0.1978; p=0.3935					
		tdT+		n=8/3 mice	KS=0.1480; p=0.8725					
							Dunn's multiple comparisons test	Sal vs tdT-	*P* =0.5867	ns
								Sal vs tdT+	*P* =0.1963	ns
								tdT- vs tdT+	*P* >0.9999	ns
Figure 7C	EPSC (% of baseline)	Sal	cell	n=10/4 mice	KS=0.1897; p=0.4606	F=4.3716 p=0.0227*	Kruskal-Wallis ANOVA	H=8.225	*P* =0.0164	*
		tdT-		n=11/6 mice	KS=0.1465; p=0.7325					
		tdT+		n=11/6 mice	KS=0.1648; p=0.6177					
							Dunn's multiple comparisons test	Sal vs tdT-	*P* >0.9999	ns
								Sal vs tdT+	*P* =0.0345	*
								tdT- vs tdT+	*P* =0.0448	*
Figure 7F	EPSC (% of baseline)	Sal	cell	n=9/4 mice	KS=0.1374; p=0.8941	F=1.1888 p=0.3243	one-way ANOVA	F=10.30	*P* =0.0008	***
		tdT-		n=8/5 mice	KS=0.2588; p=0.1672					
		tdT+		n=7/5 mice	KS=0.1368; p=0.9302					
							Tukey's multiple comparisons test	Sal vs tdT-	*P* =0.4371	ns
								Sal vs tdT+	*P* =0.0087	**
								tdT- vs tdT+	*P* =0.0008	***
Figure 8C	cell count	Sal	mouse	n=6 mice	KS=0.3333; p=0.0359*	F=7.7824 p=0.0078*	Kruskal-Wallis ANOVA	H=8.225	*P* =0.0008	***
		Veh		n=4 mice	KS=0.2988; p=0.2353					
		IEM		n=4 mice	KS=0.2525; p=0.4904					
							Dunn's multiple comparisons test	Sal vs Veh	*P* =0.0161	*
								Sal vs IEM	*P* =0.0475	*
								Veh vs IEM	*P* >0.9999	ns
Figure 8G	Rectification index	tdT+_Veh	mouse	n=6 mice	KS=0.1796; p=0.2551	F=3.8276 p=0.0082*	Mann Whitney test	U=73	*P* =0.018	*
		tdT+_IEM	mouse	n=11 mice	KS=0.0921; p=0.9278					
Figure 8J	EPSC (% of baseline)	Sal	cell	n=8/4 mice	KS=0.1806; p=0.6190	F=2.2026 p=0.1343	one-way ANOVA	F=9.261	*P* =0.0012	**
		Veh		n=8/5 mice	KS=0.1721; p=0.6925					
		IEM		n=9/5 mice	KS=0.1340; p=0.9127					
							Tukey's multiple comparisons test	Sal vs Veh	*P* =0.0008	***
								Sal vs IEM	*P* =0.0928	ns
								Veh vs IEM	*P* =0.0899	ns

Figure 8M	EPSC (% of baseline)	Sal	cell	n=8/5 mice	KS=0.2017; p=0.4398	F=0.6688 p=0.5229	one-way ANOVA	F=6.563	*P* =0.0061	**
		Veh		n=9/5 mice	KS=0.1556; p=0.7658					
		IEM		n=7/5 mice	KS=0.2034; p=0.5129					
							Tukey's multiple comparisons test	Sal vs Veh	*P* =0.0105	*
								Sal vs IEM	*P* =0.9784	ns
								Veh vs IEM	*P* =0.0215	*
Figure S5C	EPSP (% of baseline)	Sal	slice	n=12/6 mice	KS=0.1582; p=0.5573	F=0.4084 p=0.6676	one-way ANOVA	F=3.752	*P* =0.0326	*
		Veh		n=16/6 mice	KS=0.1874; p=0.1395					
		IEM		n=13/8 mice	KS=0.2179; p=0.0921					
							Tukey's multiple comparisons test	Sal vs Veh	*P* =0.0247	*
								Sal vs IEM	*P* =0.3063	ns
								Veh vs IEM	*P* =0.3452	ns
Figure S5D	LTP number	Veh	slice	n=16/6 mice			Fisher's exact test		*P* =0.0078	**
		IEM	slice	n=13/8 mice						
