# Supplementary material for: Reversible synaptic adaptations in a subpopulation of murine hippocampal neurons following early-life seizures
Source: J Clin Invest. 2024 Jan 16;134(5):e175167. doi: 10.1172/JCI175167 (PMC10904056; doi:10.1172/JCI175167)
Supplement: Supplemental table 3 [file jci-134-175167-s065.pdf]

Stat Details

Fig 1H

Linear Regression

Dependent variable	Sex
Independent variables	TRAP cell count
N	17

Regression Statistics

R	0.0207	***R-Squared***	**0.0004**	*Adjusted R-Squared*	-0.0662
MSE	0.2822	***S***	**0.5313**	*MAPE*	37.3543
Durbin-Watson (DW)	0.9470	*Log likelihood*	-12.3054		
Akaike inf. criterion (A	1.6830	*AICc*	1.6987		
Schwarz criterion (BIC	1.7810	*Hannan-Quinn criteric*	1.6927		
PRESS	5.4631	*PRESS RMSE*	0.5669	*Predicted R-Squared*	-0.2899

Sex = 1.4854 - 0.0003 * TRAP cell count

ANOVA

	d.f.	*SS*	*MS*	*F*	*p-value*
Regressio	1	0.0018	0.0018	0.0064	0.9373
Residual	15	4.2335	0.2822		
Total	16	4.2353			

Fig 1I

Linear Regression

Dependent variable	Sex
Independent variables	Td+, Duration
N	10

Regression Statistics

R	0.1077	***R-Squared***	**0.0116**	*Adjusted R-Squared*	-0.2708
MSE	0.3389	***S***	**0.5821**	*MAPE*	35.5822
Durbin-Watson (DW)	3.3220	*Log likelihood*	-6.9954		
Akaike inf. criterion (A	1.9991	*AICc*	2.1705		
Schwarz criterion (BIC	2.0899	*Hannan-Quinn criteric*	1.8995		
PRESS	8.2173	*PRESS RMSE*	0.9065	*Predicted R-Squared*	-2.4239

Sex = 1.8940 - 0.0044 * Td+ + 2.2729E-5 * Duration

ANOVA

	d.f.	*SS*	*MS*	*F*	*p-value*
Regressio	2	0.0279	0.0139	0.0411	0.9600
Residual	7	2.3721	0.3389		
Total	9	2.4000			

Fig 2C

Linear Regression

Dependent variable	Sex
Independent variables	Latnecy to seizure
N	13

Regression Statistics

R	0.2255	***R-Squared***	**0.0508**	*Adjusted R-Squared*	-0.0355
MSE	0.2788	***S***	**0.5280**	*MAPE*	35.3830
Durbin-Watson (DW)	2.7065	*Log likelihood*	-9.0576		
Akaike inf. criterion (A	1.7012	*AICc*	1.7291		
Schwarz criterion (BI(	1.7881	*Hannan-Quinn criteric*	1.6833		
PRESS	4.9833	*PRESS RMSE*	0.6191	*Predicted R-Squared*	-0.5424

Sex = 1.7217 - 0.0005 * Latnecy to seizure

ANOVA

	d.f.	*SS*	*MS*	*F*	*p-value*
Regressio	1	0.1642	0.1642	0.5891	0.4589
Residual	11	3.0665	0.2788		
Total	12	3.2308			

Fig 2D

Linear Regression

Dependent variable	Sex
Independent variables	Cumulative score
N	13

Regression Statistics

R	0.4259	***R-Squared***	**0.1814**	*Adjusted R-Squared*	0.1070
MSE	0.2404	***S***	**0.4903**	*MAPE*	30.7259
Durbin-Watson (DW)	2.6017	*Log likelihood*	-8.0954		
Akaike inf. criterion (A	1.5531	*AICc*	1.5811		
Schwarz criterion (BI(	1.6401	*Hannan-Quinn criteric*	1.5353		
PRESS	3.3656	*PRESS RMSE*	0.5088	*Predicted R-Squared*	-0.0417

Sex = 0.9300 + 0.0207 * Cumulative score

ANOVA

	d.f.	*SS*	*MS*	*F*	*p-value*
Regressio	1	0.5862	0.5862	2.4381	0.1467
Residual	11	2.6446	0.2404		
Total	12	3.2308			

Fig 2G

Linear Regression

Dependent variable	Sex
Independent variables	Colocalization
N	12

Regression Statistics

R	0.0157	***R-Squared***	**0.0002**	*Adjusted R-Squared*	-0.0997
MSE	0.2999	***S***	**0.5477**	*MAPE*	37.4908
Durbin-Watson (DW)	2.6754	*Log likelihood*	-8.7080		
Akaike inf. criterion (A	1.7847	*AICc*	1.8180		
Schwarz criterion (BI(	1.8655	*Hannan-Quinn criteric*	1.7547		
PRESS	4.3346	*PRESS RMSE*	0.6010	*Predicted R-Squared*	-0.4449

Sex = 1.4908 + 0.0002 * Colocalization

ANOVA

	d.f.	*SS*	*MS*	*F*	*p-value*
Regressio	1	0.0007	0.0007	0.0025	0.9614
Residual	10	2.9993	0.2999		
Total	11	3.0000			

Fig 3D

Linear Regression

Dependent variable	Sex
Independent variables	sEPSC freq P15
N	37

Regression Statistics

R	0.0103	***R-Squared***	**0.0001**	*Adjusted R-Squared*	-0.0285
MSE	0.2594	***S***	**0.5093**	*MAPE*	36.8113
Durbin-Watson (DW)	1.4316	*Log likelihood*	-26.5114		
Akaike inf. criterion (A	1.5412	*AICc*	1.5442		
Schwarz criterion (BIC	1.6282	*Hannan-Quinn criteric*	1.5719		
PRESS	10.2901	*PRESS RMSE*	0.5274	*Predicted R-Squared*	-0.1331

Sex = 1.5722 - 0.0111 * sEPSC freq P15

ANOVA

	d.f.	*SS*	*MS*	*F*	*p-value*
Regressio	1	0.0010	0.0010	0.0037	0.9519
Residual	35	9.0801	0.2594		
Total	36	9.0811			

Fig 3E

Linear Regression

Dependent variable	Sex
Independent variables	sEPSC amp P15
N	40

Regression Statistics

R	0.0217	***R-Squared***	**0.0005**	*Adjusted R-Squared*	-0.0258
MSE	0.2571	***S***	**0.5071**	*MAPE*	36.6391
Durbin-Watson (DW)	1.1203	*Log likelihood*	-28.5671		
Akaike inf. criterion (A	1.5284	*AICc*	1.5310		
Schwarz criterion (BIC	1.6128	*Hannan-Quinn criteric*	1.5589		
PRESS	10.9517	*PRESS RMSE*	0.5233	*Predicted R-Squared*	-0.1204

Sex = 1.6082 - 0.0011 * sEPSC amp P15

ANOVA

	d.f.	*SS*	*MS*	*F*	*p-value*
Regressio	1	0.0046	0.0046	0.0178	0.8945
Residual	38	9.7704	0.2571		
Total	39	9.7750			

Fig 3G

Linear Regression

Dependent variable	Sex
Independent variables	sEPSC freq P30
N	57

Regression Statistics

R	0.1003	***R-Squared***	**0.0101**	*Adjusted R-Squared*	-0.0079
MSE	0.2545	***S***	**0.5045**	*MAPE*	36.8371
Durbin-Watson (DW)	0.7593	*Log likelihood*	-40.8618		
Akaike inf. criterion (A	1.5039	*AICc*	1.5052		
Schwarz criterion (BIC	1.5756	*Hannan-Quinn criteric*	1.5318		
PRESS	15.2265	*PRESS RMSE*	0.5168	*Predicted R-Squared*	-0.0768

Sex = 1.4894 + 0.0731 * sEPSC freq P30

ANOVA

	d.f.	*SS*	*MS*	*F*	*p-value*
Regressio	1	0.1422	0.1422	0.5589	0.4579
Residual	55	13.9981	0.2545		
Total	56	14.1404			

Fig 3H

Linear Regression

Dependent variable	A1:A60
Independent variables	sEPSC Amp P30
N	57

Regression Statistics

R	0.1566	***R-Squared***	**0.0245**	*Adjusted R-Squared*	0.0068
MSE	0.2433	***S***	**0.4933**	*MAPE*	35.2182
Durbin-Watson (DW)	1.0757	*Log likelihood*	-39.5809		
Akaike inf. criterion (A	1.4590	*AICc*	1.4603		
Schwarz criterion (BIC	1.5307	*Hannan-Quinn criteric*	1.4868		
PRESS	14.3337	*PRESS RMSE*	0.5015	*Predicted R-Squared*	-0.0448

A1:A60 = 1.7565 - 0.0057 * sEPSC Amp P30

ANOVA

	d.f.	*SS*	*MS*	*F*	*p-value*
Regressio	1	0.3364	0.3364	1.3824	0.2447
Residual	55	13.3829	0.2433		
Total	56	13.7193			

Fig 3M

Linear Regression

Dependent variable	Sex
Independent variables	NA ratio
N	27

Regression Statistics

R	0.0646	***R-Squared***	**0.0042**	*Adjusted R-Squared*	-0.0357
MSE	0.2656	***S***	**0.5153**	*MAPE*	36.8826
Durbin-Watson (DW)	1.3700	*Log likelihood*	-19.3722		
Akaike inf. criterion (A	1.5831	*AICc*	1.5891		
Schwarz criterion (BIC	1.6791	*Hannan-Quinn criteric*	1.6117		
PRESS	7.7382	*PRESS RMSE*	0.5354	*Predicted R-Squared*	-0.1607

Sex = 1.5034 + 0.0254 * NA ratio

ANOVA

	d.f.	*SS*	*MS*	*F*	*p-value*
Regressio	1	0.0278	0.0278	0.1047	0.7489
Residual	25	6.6389	0.2656		
Total	26	6.6667			

Fig 4B

Linear Regression

Dependent variable	Sex
Independent variables	RNAscope
N	284

Regression Statistics

R	0.0566	***R-Squared***	**0.0032**	*Adjusted R-Squared*	-0.0003
MSE	0.2482	***S***	**0.4982**	*MAPE*	36.9628
Durbin-Watson (DW)	0.0603	*Log likelihood*	-204.0757		
Akaike inf. criterion (A	1.4512	*AICc*	1.4513		
Schwarz criterion (BIC	1.4769	*Hannan-Quinn criteric*	1.4615		
PRESS	71.0006	*PRESS RMSE*	0.5000	*Predicted R-Squared*	-0.0113

Sex = 1.5014 + 0.0282 * RNAscope

ANOVA

	d.f.	*SS*	*MS*	*F*	*p-value*
Regressio	1	0.2249	0.2249	0.9062	0.3419
Residual	282	69.9829	0.2482		
Total	283	70.2077			

Fig 5C

Linear Regression

Dependent variable	Sex
Independent variables	100% GluA1
N	19

Regression Statistics

R	0.2661	***R-Squared***	**0.0708**	*Adjusted R-Squared*	0.0162
MSE	0.2416	***S***	**0.4916**	*MAPE*	32.4307
Durbin-Watson (DW)	1.6863	*Log likelihood*	-12.4103		
Akaike inf. criterion (A	1.5169	*AICc*	1.5293		
Schwarz criterion (BI(	1.6163	*Hannan-Quinn criteric*	1.5337		
PRESS	5.2598	*PRESS RMSE*	0.5261	*Predicted R-Squared*	-0.1897

Sex = 2.2217 - 0.0045 * 100% GluA1

ANOVA

	d.f.	*SS*	*MS*	*F*	*p-value*
Regressio	1	0.3132	0.3132	1.2960	0.2707
Residual	17	4.1079	0.2416		
Total	18	4.4211			

Fig 5F

Linear Regression

Dependent variable	Sex
Independent variables	100% GluA2
N	19

Regression Statistics

R	0.2942	***R-Squared***	**0.0866**	*Adjusted R-Squared*	0.0328
MSE	0.2376	***S***	**0.4874**	*MAPE*	31.9277
Durbin-Watson (DW)	2.0882	*Log likelihood*	-12.2481		
Akaike inf. criterion (A	1.4998	*AICc*	1.5122		
Schwarz criterion (BI(	1.5992	*Hannan-Quinn criteric*	1.5166		
PRESS	4.8606	*PRESS RMSE*	0.5058	*Predicted R-Squared*	-0.0994

Sex = 0.8508 + 0.0075 * 100% GluA2

ANOVA

	d.f.	*SS*	*MS*	*F*	*p-value*
Regressio	1	0.3827	0.3827	1.6110	0.2215
Residual	17	4.0384	0.2376		
Total	18	4.4211			

Fig 5K

Linear Regression

Dependent variable	Sex
Independent variables	pGluA2 density
N	296

Regression Statistics

R	0.0581	***R-Squared***	**0.0034**	*Adjusted R-Squared*	########
MSE	0.2508	***S***	**0.5008**	*MAPE*	37.3666
Durbin-Watson (DW)	0.1147	*Log likelihood*	-214.3068		
Akaike inf. criterion (A	1.4615	*AICc*	1.4616		
Schwarz criterion (BI(	1.4865	*Hannan-Quinn criteric*	1.4715		
PRESS	74.8013	*PRESS RMSE*	0.5027	*Predicted R-Squared*	-0.0110

Sex = 1.5566 - 0.0003 * pGluA2 density

ANOVA

	d.f.	*SS*	*MS*	*F*	*p-value*
Regressio	1	0.2498	0.2498	0.9958	0.3191
Residual	294	73.7367	0.2508		
Total	295	73.9865			

Fig 6D

Linear Regression

Dependent variable	Sex
Independent variables	Silent Synapse
N	19

Regression Statistics

R	0.2763	***R-Squared***	**0.0764**	*Adjusted R-Squared*	0.0220
MSE	0.2574	***S***	**0.5073**	*MAPE*	34.5406
Durbin-Watson (DW)	1.1073	*Log likelihood*	-13.0091		
Akaike inf. criterion (A	1.5799	*AICc*	1.5923		
Schwarz criterion (BIC	1.6793	*Hannan-Quinn criteric*	1.5967		
PRESS	5.4672	*PRESS RMSE*	0.5364	*Predicted R-Squared*	-0.1542

Sex = 1.8157 - 0.6864 * Silent Synapse

ANOVA

	d.f.	*SS*	*MS*	*F*	*p-value*
Regressio	1	0.3617	0.3617	1.4054	0.2521
Residual	17	4.3751	0.2574		
Total	18	4.7368			

Fig 6H

Linear Regression

Dependent variable	Sex
Independent variables	Silent Synapse (P30)
N	26

Regression Statistics

R	0.0295	***R-Squared***	**0.0009**	*Adjusted R-Squared*	-0.0408
MSE	0.2306	***S***	**0.4802**	*MAPE*	31.9249
Durbin-Watson (DW)	0.7427	*Log likelihood*	-16.7781		
Akaike inf. criterion (A	1.4445	*AICc*	1.4509		
Schwarz criterion (BIC	1.5412	*Hannan-Quinn criteric*	1.4723		
PRESS	6.8932	*PRESS RMSE*	0.5149	*Predicted R-Squared*	-0.2446

Sex = 1.6636 + 0.1325 * Silent Synapse (P30)

ANOVA

	d.f.	*SS*	*MS*	*F*	*p-value*
Regressio	1	0.0048	0.0048	0.0209	0.8862
Residual	24	5.5336	0.2306		
Total	25	5.5385			

Fig 7C

Linear Regression

Dependent variable	Sex
Independent variables	LTP
N	30

Regression Statistics

R	0.0125	***R-Squared***	**0.0002**	*Adjusted R-Squared*	-0.0356
MSE	0.2631	***S***	**0.5129**	*MAPE*	36.8275
Durbin-Watson (DW)	0.9532	*Log likelihood*	-21.5023		
Akaike inf. criterion (A	1.5668	*AICc*	1.5716		
Schwarz criterion (BIC	1.6602	*Hannan-Quinn criteric*	1.5967		
PRESS	8.5303	*PRESS RMSE*	0.5332	*Predicted R-Squared*	-0.1580

Sex = 1.4470 - 0.0001 * LTP last 10 min

ANOVA

	d.f.	*SS*	*MS*	*F*	*p-value*
Regressio	1	0.0012	0.0012	0.0044	0.9476
Residual	28	7.3655	0.2631		
Total	29	7.3667			

Fig 7F

Linear Regression

Dependent variable	Sex
Independent variables	LTD
N	24

Regression Statistics

R	0.0701	***R-Squared***	**0.0049**	*Adjusted R-Squared*	-0.0403
MSE	0.2695	***S***	**0.5191**	*MAPE*	37.0564
Durbin-Watson (DW)	1.5378	*Log likelihood*	-17.2762		
Akaike inf. criterion (A	1.6063	*AICc*	1.6139		
Schwarz criterion (BIC	1.7045	*Hannan-Quinn criteric*	1.6324		
PRESS	7.1111	*PRESS RMSE*	0.5443	*Predicted R-Squared*	-0.1935

Sex = 1.3745 + 0.0013 * LTD last 10 min

ANOVA

	d.f.	*SS*	*MS*	*F*	*p-value*
Regressioı	1	0.0293	0.0293	0.1088	0.7447
Residual	22	5.9290	0.2695		
Total	23	5.9583			

Fig 8C

Linear Regression

Dependent variable	Sex
Independent variables	Cell count
N	14

Regression Statistics

R	0.0347	***R-Squared***	**0.0012**	*Adjusted R-Squared*	-0.0820
MSE	0.2675	***S***	**0.5172**	*MAPE*	34.3972
Durbin-Watson (DW)	2.1912	*Log likelihood*	-9.5565		
Akaike inf. criterion (A	1.6509	*AICc*	1.6747		
Schwarz criterion (BIC	1.7422	*Hannan-Quinn criteric*	1.6425		
PRESS	4.3520	*PRESS RMSE*	0.5575	*Predicted R-Squared*	-0.3539

Sex = 1.6218 + 0.0006 * Cell count

ANOVA

	d.f.	*SS*	*MS*	*F*	*p-value*
Regressioı	1	0.0039	0.0039	0.0145	0.9062
Residual	12	3.2104	0.2675		
Total	13	3.2143			

Fig 8G

Linear Regression

Dependent variable	Sex
Independent variables	RI IEM at P30
N	34

Regression Statistics

R	0.0529	***R-Squared***	**0.0028**	*Adjusted R-Squared*	-0.0284
MSE	0.2612	***S***	**0.5111**	*MAPE*	36.8773
Durbin-Watson (DW)	0.9478	*Log likelihood*	-24.3923		
Akaike inf. criterion (A	1.5525	*AICc*	1.5562		
Schwarz criterion (BIC	1.6423	*Hannan-Quinn criteric*	1.5831		
PRESS	9.5084	*PRESS RMSE*	0.5288	*Predicted R-Squared*	-0.1343

Sex = 1.6044 - 0.0263 * RI IEM at P30

ANOVA

	d.f.	*SS*	*MS*	*F*	*p-value*
Regressioı	1	0.0235	0.0235	0.0899	0.7662
Residual	32	8.3589	0.2612		
Total	33	8.3824			

Fig 8J	**Linear Regression**

Dependent variable	Sex
Independent variables	IEM LTP
N	25

Regression Statistics

R	0.1681	***R-Squared***	**0.0283**	*Adjusted R-Squared*	-0.0140
MSE	0.2603	***S***	**0.5102**	*MAPE*	35.9153
Durbin-Watson (DW)	1.2566	*Log likelihood*	-17.6051		
Akaike inf. criterion (A	1.5684	*AICc*	1.5754		
Schwarz criterion (BIC	1.6659	*Hannan-Quinn criteric*	1.5954		
PRESS	7.0308	*PRESS RMSE*	0.5303	*Predicted R-Squared*	-0.1414

Sex = 1.1585 + 0.0032 * IEM LTP last 10 min

ANOVA

	d.f.	*SS*	*MS*	*F*	*p-value*
Regressioı	1	0.1741	0.1741	0.6690	0.4218
Residual	23	5.9859	0.2603		
Total	24	6.1600			

Fig 8M	**Linear Regression**

Dependent variable	Sex
Independent variables	IEM LTD
N	24

Regression Statistics

R	0.1899	***R-Squared***	**0.0361**	*Adjusted R-Squared*	-0.0077
MSE	0.2611	***S***	**0.5109**	*MAPE*	35.8962
Durbin-Watson (DW)	1.7839	*Log likelihood*	-16.8945		
Akaike inf. criterion (A	1.5745	*AICc*	1.5821		
Schwarz criterion (BIC	1.6727	*Hannan-Quinn criteric*	1.6006		
PRESS	7.0804	*PRESS RMSE*	0.5432	*Predicted R-Squared*	-0.1883

Sex = 1.3151 + 0.0038 * IEM LTD last 10 min

ANOVA

	d.f.	*SS*	*MS*	*F*	*p-value*
Regressioı	1	0.2149	0.2149	0.8233	0.3740
Residual	22	5.7434	0.2611		
Total	23	5.9583			

Fig S5C	**Linear Regression**

Dependent variable	Sex
Independent variables	MEA LTP
N	41

Regression Statistics

R	0.1686	***R-Squared***	**0.0284**	*Adjusted R-Squared*	0.0035
MSE	0.2552	***S***	**0.5052**	*MAPE*	36.4122
Durbin-Watson (DW)	0.9711	*Log likelihood*	-29.1540		
Akaike inf. criterion (A	1.5197	*AICc*	1.5222		
Schwarz criterion (BIC	1.6033	*Hannan-Quinn criteric*	1.5501		
PRESS	10.9987	*PRESS RMSE*	0.5179	*Predicted R-Squared*	-0.0737

Sex = 1.8665 - 0.3149 * MEA last 10 min

ANOVA

	d.f.	*SS*	*MS*	*F*	*p-value*
Regressioı	1	0.2912	0.2912	1.1412	0.2920
Residual	39	9.9527	0.2552		
Total	40	10.2439			
